# Biofilm Formation As a Response to Ecological Competition

**DOI:** 10.1371/journal.pbio.1002191

**Published:** 2015-07-09

**Authors:** Nuno M. Oliveira, Esteban Martinez-Garcia, Joao Xavier, William M. Durham, Roberto Kolter, Wook Kim, Kevin R. Foster

**Affiliations:** 1 Department of Zoology, University of Oxford, Oxford, United Kingdom; 2 Oxford Centre for Integrative Systems Biology, University of Oxford, Oxford, United Kingdom; 3 FAS Center for Systems Biology, University of Harvard, Cambridge, Massachusetts, United States of America; 4 Centro Nacional de Biotecnologia-CSIC, Campus de Cantoblanco, Madrid, Spain; 5 Memorial Sloan-Kettering Cancer Center, Computational Biology Program, New York, New York, United States of America; 6 Harvard Medical School, Department of Microbiology and Immunobiology, Boston, Massachusetts, United States of America; Massachusetts Institute of Technology, United States

## Abstract

Bacteria form dense surface-associated communities known as biofilms that are central to their persistence and how they affect us. Biofilm formation is commonly viewed as a cooperative enterprise, where strains and species work together for a common goal. Here we explore an alternative model: biofilm formation is a response to ecological competition. We co-cultured a diverse collection of natural isolates of the opportunistic pathogen *Pseudomonas aeruginosa* and studied the effect on biofilm formation. We show that strain mixing reliably increases biofilm formation compared to unmixed conditions. Importantly, strain mixing leads to strong competition: one strain dominates and largely excludes the other from the biofilm. Furthermore, we show that pyocins, narrow-spectrum antibiotics made by other *P*. *aeruginosa* strains, can stimulate biofilm formation by increasing the attachment of cells. Side-by-side comparisons using microfluidic assays suggest that the increase in biofilm occurs due to a general response to cellular damage: a comparable biofilm response occurs for pyocins that disrupt membranes as for commercial antibiotics that damage DNA, inhibit protein synthesis or transcription. Our data show that bacteria increase biofilm formation in response to ecological competition that is detected by antibiotic stress. This is inconsistent with the idea that sub-lethal concentrations of antibiotics are cooperative signals that coordinate microbial communities, as is often concluded. Instead, our work is consistent with competition sensing where low-levels of antibiotics are used to detect and respond to the competing genotypes that produce them.

## Abstract 2

Mixing natural isolates of the pathogenic bacterium *Pseudomonas aeruginosa* shows that the formation of biofilm is a response to antibiotic stress from competing genotypes.

## Author Summary

Bacteria often attach to each other and to surfaces and make biofilms. These dense communities occur everywhere, including on us and inside us, where they are central to both health and disease. Biofilm formation is often viewed as the coordinated action of multiple strains that work together in order to prosper and protect each other. In this study, we provide evidence for a very different view: biofilms are formed when bacterial strains compete with one another. We mixed together different strains of the widespread pathogen *Pseudomonas aeruginosa* and found that pairs often make bigger biofilms than either one alone. Rather than working together, however, we show that one strain normally kills the other off and that biofilm formation is actually a response to the damage of antibiotic warfare. Our work helps to explain the widespread observation that treating bacteria with clinical antibiotics can stimulate biofilm formation. When we treat bacteria, they respond as if the attack is coming from a foreign strain that must be outnumbered and outcompeted in a biofilm.

## Introduction

While the traditional model for bacterial life was one of cells swimming in liquid, it is now realized that bacteria commonly live in surface-associated communities known as biofilms [1–4]. These groups of bacteria carry significant medical and economic importance that includes a role in chronic diseases, antibiotic tolerance, biofouling, and waste-water treatment [5–7]. The importance of biofilms has led to their intensive study in many species of bacteria. However, this work has revealed that the genetic and biochemical mechanisms underlying biofilm formation are extremely variable across strains and growth conditions [8,9]. This variability has made it difficult to identify common principles of biofilm formation across species.

One property common to the biofilms of many bacterial species, both gram-negative and gram-positive, is that they are induced by sub-lethal concentrations of antibiotics [10–17]. The diversity of the species displaying this response is striking [13] and suggestive of the general principles that so often elude the study of biofilms. This, and the observation that sub-lethal antibiotics induce a range of other physiological changes [18], has led to the hypothesis that antibiotics may not function as killing agents in nature. Instead, it is suggested that antibiotics may function as cooperative signals between species that contribute to the homeostasis of bacterial communities [13,16,19–21]. In parallel, there are a growing number of studies concluding that natural microbial communities are cooperative enterprises in which strains and species work together, both in biofilm formation and metabolism [22–31].

Cooperative phenotypes—such as secreted enzymes or polymers—are important in biofilms for cells of a single genotype [4,32–35]. However, evolutionary theory and ecological experiments caution against the idea that this cooperation will always extend to cells of different genotypes [32,34,36–38]. Competition between strains and species appears to be commonplace and it is not clear how cooperative signaling via antibiotics could widely evolve [39]. An alternative to the idea that sub-lethal antibiotics are cooperative signals between species is that they are cues used by competing species to detect competitors and respond appropriately [39–41]. Under the cue model, antibiotics are not secreted in order to signal to others [42,43], they are secreted to attack but can inadvertently provide information to others at low concentration. The idea that bacteria use antibiotics and other factors as cues of competition is central to the idea of “competition sensing” [41]. This is based on the observation that both nutrient and antibiotic stress, which are associated with ecological competition, often cause bacteria to release their own antibiotics. Bacteria appear to attack back when they are themselves attacked.

If sub-lethal concentrations of antibiotics are cues of competition from other strains, and biofilms are induced by such concentrations, this implies that biofilm formation can be a response to ecological competition from other strains. Here we test this hypothesis by co-culturing natural isolates of the opportunistic pathogen *Pseudomonas aeruginosa* and studying the effects on biofilm formation. We focus on members of a single species here because these are expected to have the strongest ecological overlap and, therefore, the strongest ecological competition; something first noted by Darwin: “The struggle almost invariably will be most severe between the individuals of the same species, for they frequent the same districts, require the same food, and are exposed to the same dangers” [44]. More concretely, data from the cystic fibrosis lung suggest that different isolates can colonize and, at least temporarily, co-exist in young patients [45–47], which is consistent with different strains meeting. *P*. *aeruginosa* also forms robust biofilms that have been intensively studied [6,48]. The co-culture of *P*. *aeruginosa* strains then provides a test case to evaluate the link between ecological competition and biofilm formation. Specifically, if biofilm formation is a response to ecological competition, we expect to observe (i) evidence of competition between strains and (ii) evidence of increased biofilm formation in co-cultures relative to monocultures.

## Results and Discussion

### Both Clinical Antibiotics and Mixing Strains Promote Biofilm Formation

**Fig 1:**
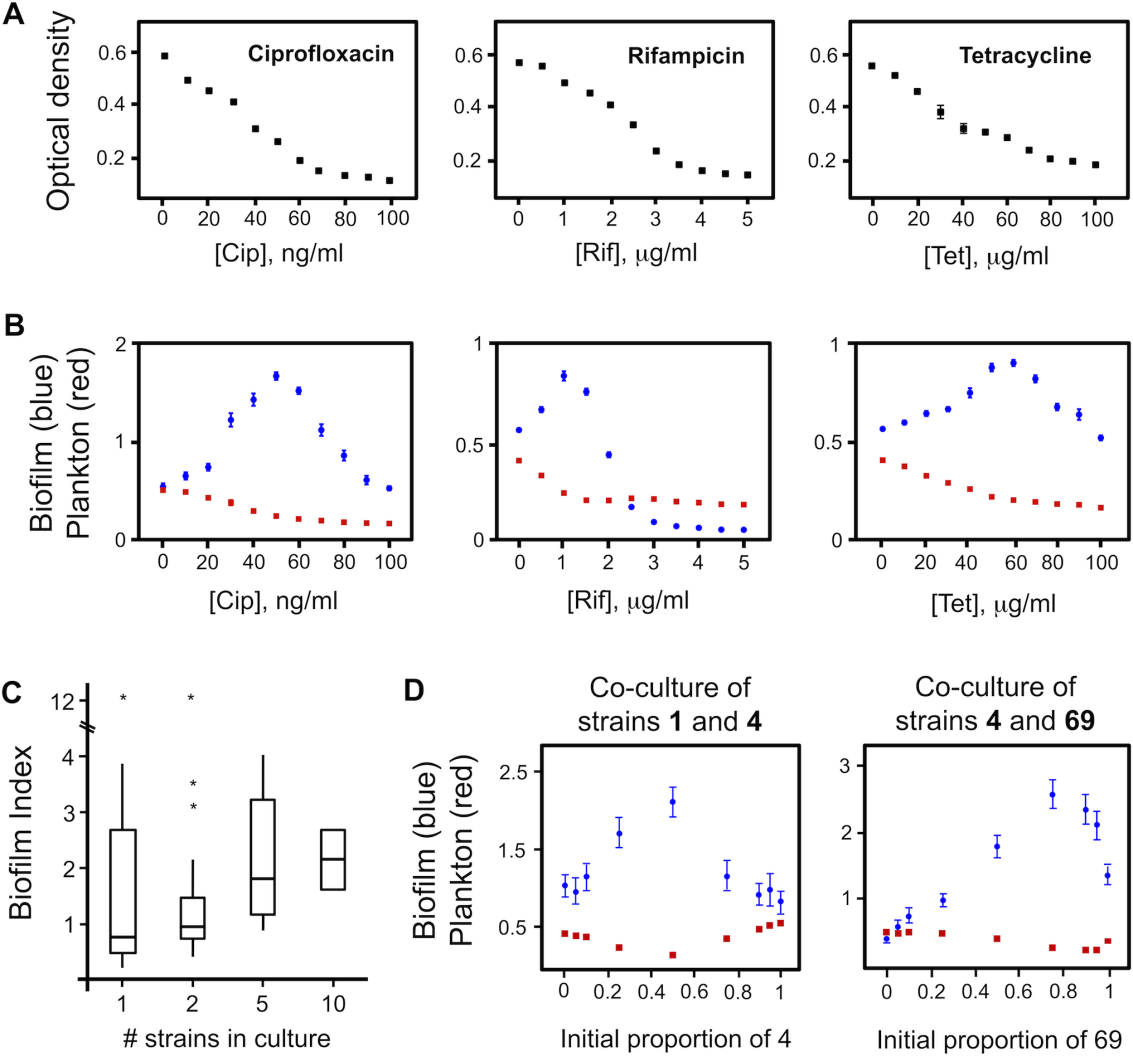
Effect of clinical antibiotics and strain mixing on biofilm formation of *P*. *aeruginosa*. A) Increasing concentrations of three antibiotics from different classes, ciprofloxacin (Cip), rifampicin (Rif), and tetracycline (Tet), reduce the optical density of strain PAO1 shaking cultures. B) Under static conditions, sub-lethal concentrations of antibiotics induce biofilm formation. C) Mixing different natural isolates of *P*. *aeruginosa* induces biofilm formation in an assay with increasing numbers of strains. The *y*-axis of box plots represents the biofilm index of each strain and mixtures, which is the ratio of biofilm to planktonic cell density (A_595_/A_600_). This measure controls for the large variability in overall growth between the strains and is a way to assess biofilm relative to the amount of planktonic cells [49]. The biofilm index increases with the more strains present (2-tailed Spearman rank correlation, non-normal data, *n* = 52, ρ = 0.305, *p* = 0.025). D) Mixing increases biofilm formation in two pairwise combinations of strains (1 + 4 and 4 + 69) in which the initial proportion of each strain was varied. Panels A, B, and D show means and 95% confidence intervals. Error bars are too small to see in some cases, particularly for the planktonic data. Find numerical values in S1 Data.

We begin by recapitulating the known effects of antibiotic stress on bacterial survival and biofilm formation. Fig 1 (panels A, B) shows the effects of antibiotics on *Pseudomonas aeruginosa* where we use optical density as a qualitative indicator of cell number. As expected, increasing the concentration of three widely used clinical antibiotics—ciprofloxacin, rifampicin and tetracycline—monotonically reduces the optical density of shaking cultures. However, as previously shown, this monotonic decrease is not seen when the same concentrations are applied to standing cultures. In standing cultures, the liquid is not agitated and cells can readily attach to the edge of the well and establish biofilms. Under this condition, biofilm formation actually increases for many concentrations of the antibiotics, until the concentrations become so high that toxicity dominates [13].

Applying sub-lethal concentrations of antibiotics then leads to an increase in biofilm formation. Can we understand these widely reported biofilm responses to antibiotics in terms of ecological competition? We tested this by mixing natural isolates of *P*. *aeruginosa* in standing cultures and studying the impact on biofilm formation. Consistent with the hypothesis that biofilm formation is a response to ecological competition, we find that biofilm formation is increased in mixed cultures compared to pure cultures, both in a broad scale survey that mixed combinations of 22 natural isolates (Fig 1C) and in defined pairwise comparisons in which we show that the strength of the biofilm response depends on the relative proportion of each strain at the start of the co-cultures (Fig 1D).

### Competitive Exclusion Is Common in Pairwise Mixtures

**Fig 2:**
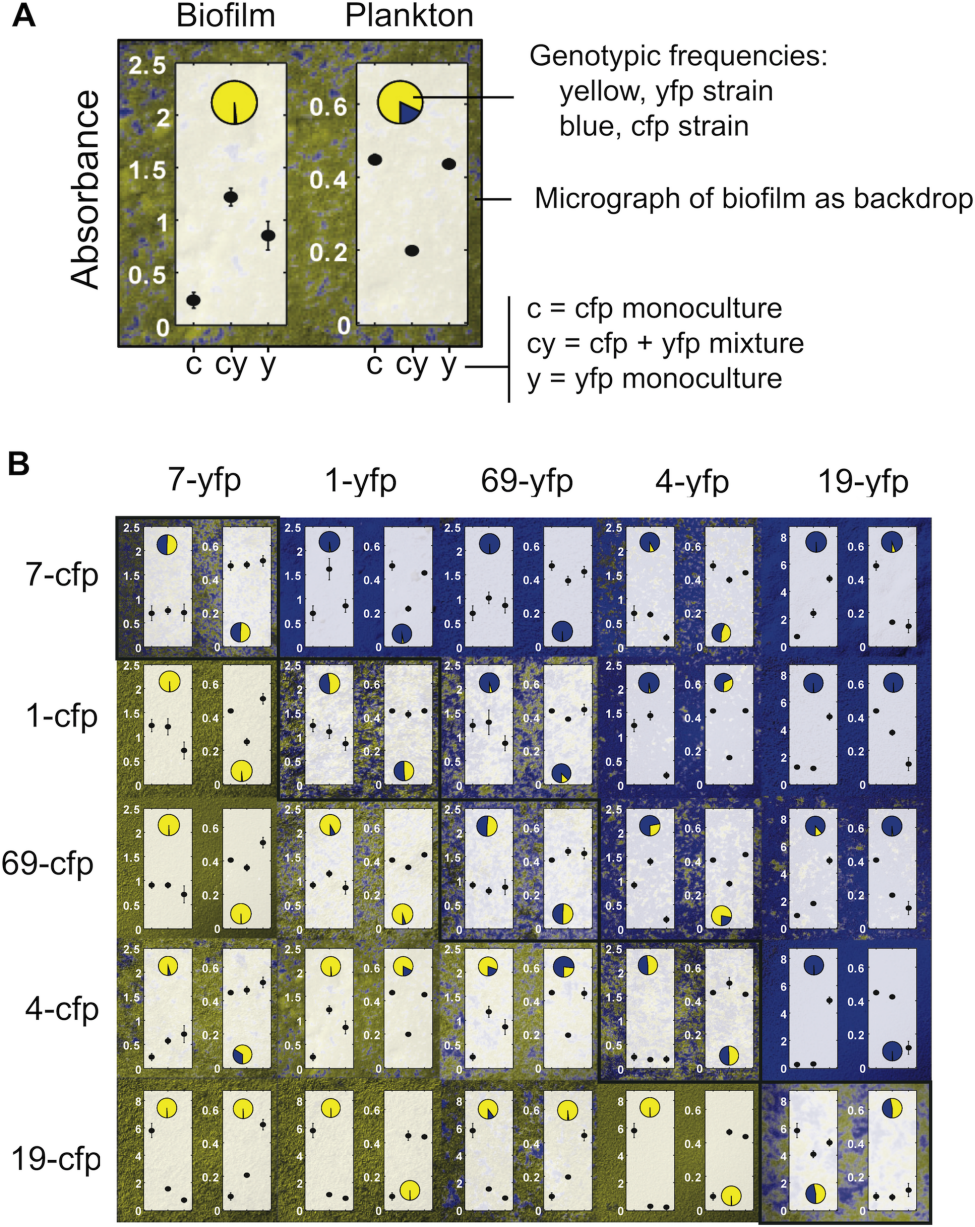
Round-robin tournament among natural isolates of *P*. *aeruginosa* showing strong competitive effects upon strain mixing. Five isolates were mixed together in all pairwise combinations and data from each mixture are shown in a corresponding panel. A) Example data from strains 4 and 1 illustrating the different measures in a given mixture. In this example, mixing the two strains increases biofilm, decreases planktonic cells, and strain 1 (*yfp*-labeled) strain strongly outcompetes strain 4 (*cfp*-labeled) strain. B) All data from the round-robin tournament. As in panel A, the backdrop to each panel is an epifluorescence image of the coculture biofilm from a coverslip. All other data shown are from 96-well microtiter experiments. Specifically, the left-hand side graph in each panel shows information about the biofilm. Here, the plotted points show biofilm formation in the crystal violet assay by the *cfp*-labeled strain alone, both strains together, and the *yfp*-labeled strain alone, from left to right. The pie chart on the left shows frequencies of the two strains in the biofilm. The right-hand side of each panel—graph and pie chart—shows the planktonic cell data from the same experiments as the biofilms measurements. *cfp* and *yfp* stand for cyan and yellow fluorescent protein, respectively. Plotted data show means and 95% confidence intervals. Find numerical values in S1 Data.

The observed biofilm response is consistent with a competitive response. However, an alternative hypothesis is that the strains cooperate with one another to produce more biofilm (see Introduction). In order to better assess whether the strains were cooperating to make more biofilm, we labeled five of our *P*. *aeruginosa* natural isolates with constitutive fluorescent proteins (cyan fluorescent protein, CFP, and yellow fluorescent protein, YFP) by putting a single copy of the desired fluorescent protein gene at a specific site in the genome [50]. We then set up a round-robin tournament, in which the five strains were mixed in all combinations, and measured biofilm formation, planktonic growth, and the relative frequency of the two strains (Fig 2). This revealed a strong competitive effect with one strain typically dominating after 24 h (median proportion of dominant strain in biofilm = 0.98, Wilcoxon-signed rank test versus 0.5, *n* = 10, *p* < 0.01). Thus, the biofilm response is a manifestation of competition between strains rather than a cooperative synergy in which both strains benefit from the presence of one another [34].

There are some cases in which biofilm formation in the mixture is less than that expected from the average of the two starting strains (Fig 2). However, accounting for which strain actually makes the final biofilm reveals that mixing nearly always causes an increase in biofilm formation by the dominant strain. For example, mixtures of 7 and 19 result in less biofilm than monocultures of strain 19. However, strain 7 eliminates strain 19 in co-cultures, producing more biofilm than it does when alone (Fig 2). More generally, we can calculate the expected biofilm formation from the pure culture data using a weighted average, which weights by the frequencies of the two strains in the biofilm after competition. This reveals that the mixed culture biofilm normally increases relative to the expectation based upon pure cultures (9/10 mixes increase, Wilcoxon signed rank test of expected/observed biofilm against a null of 1, *n* = 10, *p* < 0.01), while the amount of cells in the planktonic phase decreases (*n* = 10, *p* < 0.01). Moreover, the increases in biofilm and decreases in plankton that we observe upon mixing strains are significantly negatively correlated across the different strain mixes (Spearman rank correlation, *n* = 10, *r* = -0.782, *p* < 0.01). This correlation agrees with recent observations on multispecies interactions during biofilm formation [31].

The biofilm response is not an idiosyncrasy of our particular growth medium. S1 Fig shows the effect of varying levels of nutrients, buffering, and soluble iron for two genotype mixtures. Despite the success of strains in pairwise competition being strongly dependent on the environment, the biofilm response is detected across all conditions in at least one of the two strain combinations, but typically in both (S1 Fig).

### Pyocins Serve As a Cue for Biofilm Formation

**Fig 3:**
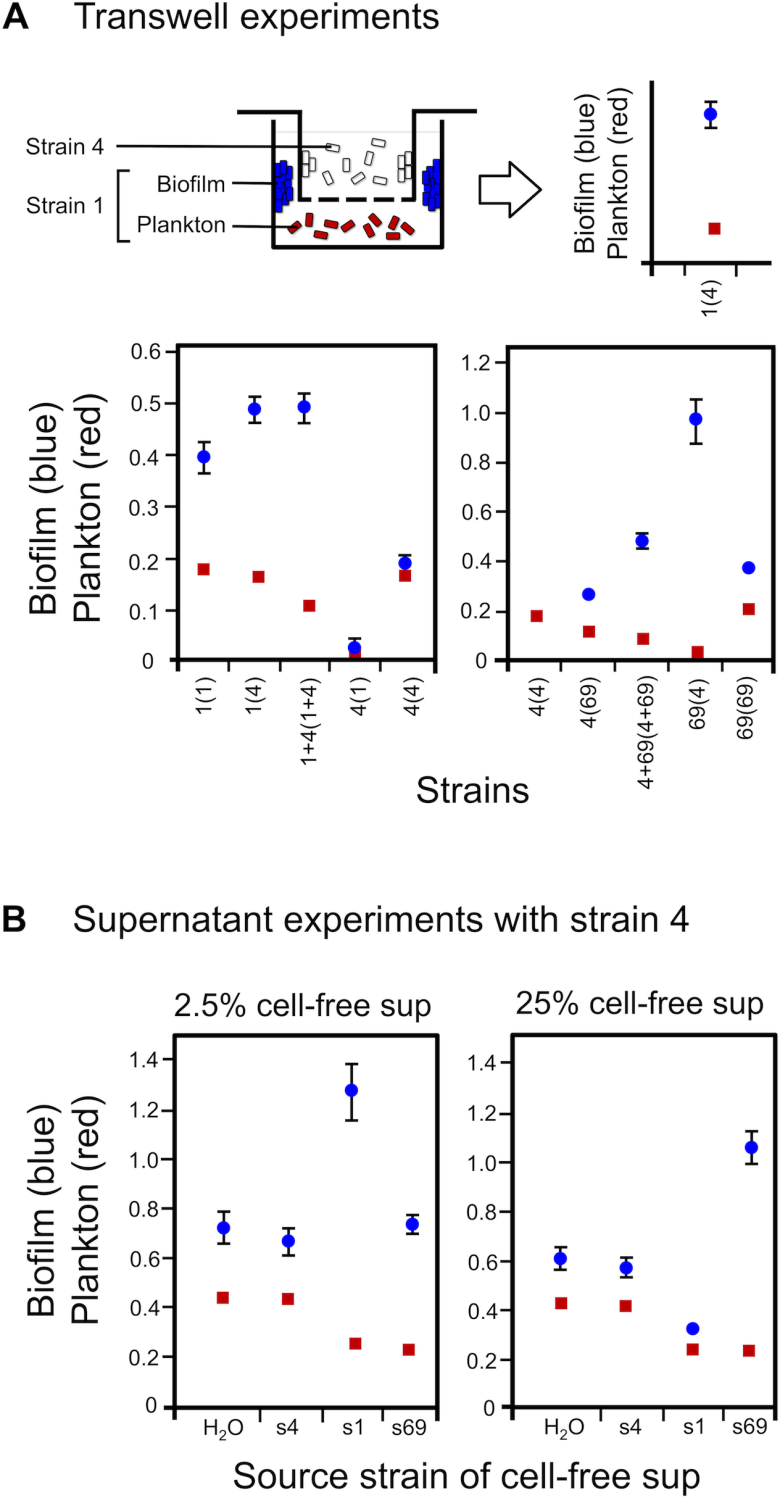
Increased biofilm in co-cultures is not dependent on cellular contact between strains. A) Membrane-separated growth using Transwell plates (see Methods). Top diagram shows experimental design (left) and how results are plotted (right). On the axes, the first number refers to the responder strain for which the data are shown, while the number in parentheses is the inducer strain that is grown above, across the membrane. B) Increased biofilm due to cell-free culture supernatants. Data are for strain 4 growing in the presence of various supernatants (“H_2_O” is water in place of the supernatant, and “s4,” “s1,” and “s69” indicate the supernatant from strains 4, 1, and 69, respectively). Two concentrations of the supernatant are shown: 2.5% (left) and 25% (right). Data show means and 95% confidence intervals. Find numerical values in S1 Data.

We next focus on the cue that drives the increase in biofilm formation in co-cultures. We first examined whether the biofilm response requires cell–cell contact by growing pairs of strains on either side of a 0.4 μm permeable membrane (Fig 3A). Increased biofilm is observed across the membrane in most cases. In one case, there is a strong inhibitory effect on both biofilm and planktonic growth, which is consistent with the responder strain losing in co-culture (specifically, strain 4 is inhibited and outcompeted by strain 1, Fig 2). We also found that the biofilm response is recapitulated by the addition of cell-free culture supernatant of different strains. We focus on strain 4, which responds robustly to the addition of the supernatant from other strains (Fig 3B). The nature of the response is dose dependent: lower concentrations tend to induce biofilm formation while high concentrations inhibit both planktonic growth and biofilm formation.

**Fig 4:**
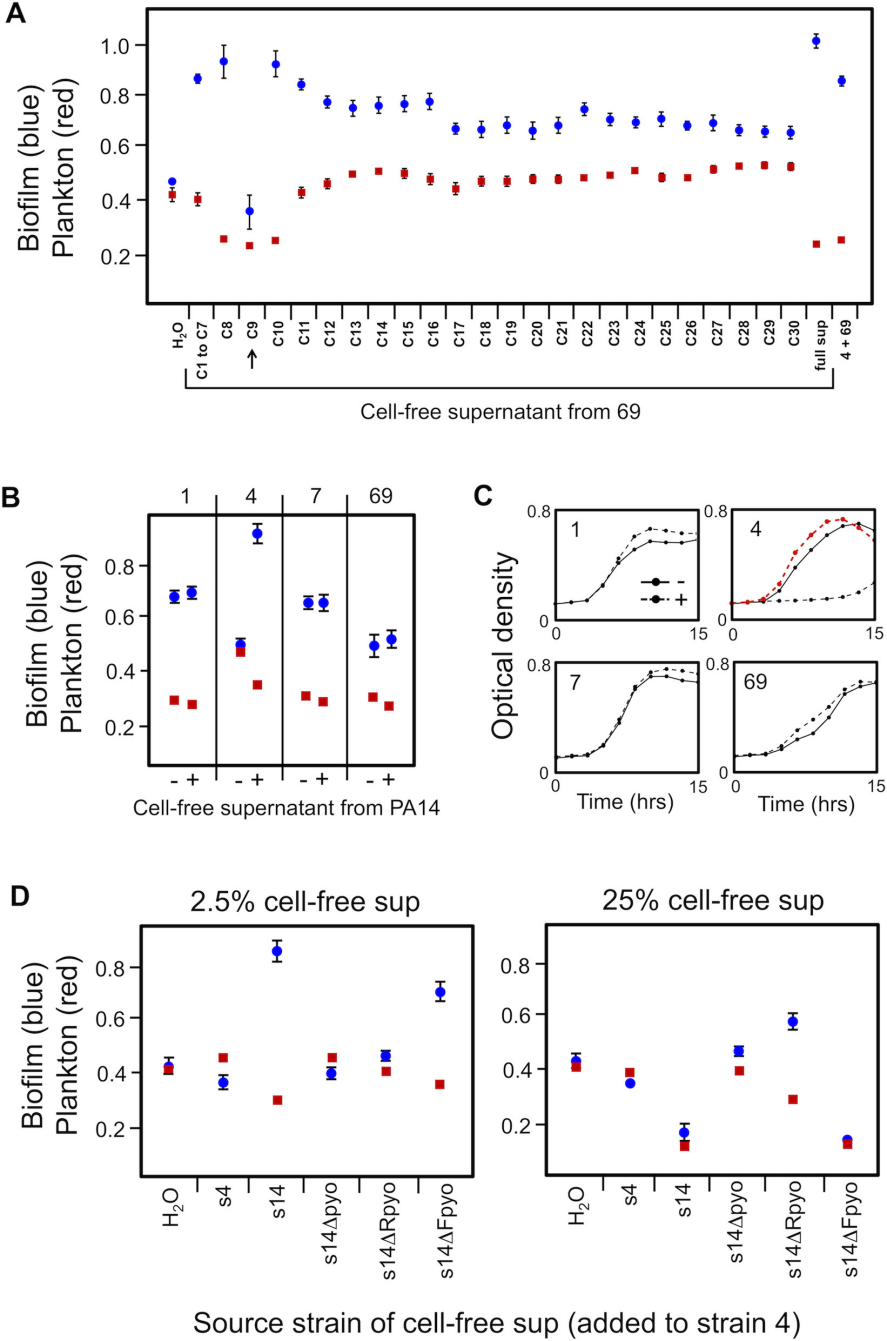
Pyocins are drivers of biofilm formation in co-cultures. A) Size-based separation of the cell-free culture supernatant from strain 69 isolates fractions that induce biofilm formation at low concentrations and growth inhibition at high concentrations. Adding the unfractionated supernatant (“full sup”) induces biofilm in strain 4, which is comparable to direct mixtures of strains 4 and 69 (here labeled as “4 + 69”). Mass spectrometry analysis of fraction C9 (arrow) identified three proteins that are homologous to R and F pyocins in strain PA14. Compared to manufacturer size standards for the column, fraction C9 is expected to be >600 kDa. B) Cell-free culture supernatant at 2.5% in tryptone broth from PA14 (“+”) causes increased biofilm formation in strain 4 relative to tryptone broth with the same amount of water added (“-”), but this effect is not seen for strains 1, 7, or 69. C) The supernatant from PA14 is toxic to only strain 4, causing a strong population growth lag. This suggests that some degree of cell damage is needed for the biofilm response. Supernatant from a PA14 pyocin-null mutant (PA14 *prtR* ^*S153A*^) does not cause any growth lag in strain 4 (red curve), confirming that the toxicity to strain 4 is driven by pyocins. D) R pyocins, and F pyocins to a lesser degree, drive the biofilm response in strain 4. At 2.5%, the supernatant from both PA14 (“s14”) and F-pyocin-null mutant (“s14ΔFpyo”) elicit a strong biofilm response in strain 4, which is largely removed by mutating either a master regulator of pyocins (“s14Δpyo”) or R pyocins (“s14ΔRpyo”) in PA14 (left panel). At a higher concentration (25%), supernatant that contains R pyocins is strongly toxic to strain 4, and R pyocin free supernatant now induces biofilm formation, which is consistent with the effects of other pyocins. Data from biofilm assays are means and 95% confidence intervals. Growth curves are means of eight replicates. Find numerical values in S1 Data.

In order to identify the active component within the supernatant, we first performed anion exchange chromatography on the supernatant of strain 69 as it generates a robust response in strain 4 (Fig 3B). The active fraction that induced biofilm formation in strain 4 was further separated based on size by gel filtration. A single fraction (C9) appeared to contain the bulk of the active component as it strongly inhibited growth in strain 4, while the two flanking fractions C8 and C10 (i.e., lower concentrations of the effector) induced the biofilm response (Fig 4A). We subjected fraction C9 to protein mass spectrometry (see Methods). Of the proteins identified, three are notable for their known role in microbial interactions: homologs (PA14_8070, PA14_8090, PA14_8210) of the R and F pyocins in *P*. *aeruginosa* strain PA14 (S1 Table) [51]. Given that PA14 is a well-characterized strain, we next sought to explore how the pyocins of PA14 induce the biofilm response in our natural isolates.

We first evaluated the effects of cell-free culture supernatant from PA14 on biofilm formation in the five strains from the round robin tournament. Strain 19, which was isolated from a cystic fibrosis infection, is a very poor competitor (Fig 2) and showed no detectable growth in our supernatant assays. It was therefore excluded from further analysis. Among the remaining strains, increased biofilm was observed in only strain 4 (Fig 4B), and we confirmed that direct mixtures of PA14 and strain 4 also lead to increased biofilm formation (S2 Fig). Why is the biofilm response absent in the other three strains? To address this question, we grew the four strains in shaking culture in the presence or absence of the supernatant from PA14 and followed their growth over time. The results obtained show that the strains that do not show the biofilm response to the supernatant of PA14 also are not harmed by it (Fig 4C). This correlation between toxicity and increased biofilm helps explain the lack of response in the three strains. Cell damage appears to be required for the biofilm response.

We next studied the effect of various mutants of pyocin production in PA14 on their ability to damage and induce the biofilm response in strain 4. We focused particularly on a strain of PA14 that has the repressor of pyocins, *prtR*, mutated to a version that is constitutively active (S3 Table) [52]. We added cell-free supernatant of the PA14 *prtR* mutant to strain 4 and compared the effect to the addition of WT PA14 supernatant by propidium iodide staining. Propidium iodide preferentially enters cells with envelope damage (see Methods), and this assay confirmed that the pyocins produced by PA14 cause cell damage to strain 4 (S3 Fig), which agrees with published literature on pyocin-driven lethality [51]. Moreover, additional experiments showed that the pyocins of PA14 are the major effectors of both population growth inhibition (Fig 4C) and the biofilm response of strain 4 (Fig 4D).

Focusing on the biofilm response, the use of mutants that lack only one class of pyocin suggested that F pyocins have some effect on biofilm formation at high concentrations, but it is particularly the R pyocins that strongly stimulate biofilm formation. This is, furthermore, consistent with the importance of cell damage because R pyocins are known to be efficient killers that can contribute significantly to competition among *P*. *aeruginosa* strains [53,54]. To further explore the potential of cell damage being required for the biofilm response, we screened 12 natural isolates for inhibition by the supernatant of PA14 in shaking culture and found that three strains were inhibited like strain 4. We randomly selected two of the inhibited strains for further study in the biofilm assay. This revealed that both exhibit a biofilm response comparable to that of strain 4 and, again, the response occurs in a pyocin-dependent manner (S3 Fig). We also tested the response of the strain PAK under the same conditions. This serves as a good control case since PAK is known to be inhibited by the pyocins produced by PA14 [53]. Again, we find evidence of pyocin-driven biofilm formation (S4 Fig).

These data further support the idea that pyocin-driven damage under these conditions is sufficient to drive the biofilm response. We note, however, that this does not exclude the possibility that there are additional mechanisms by which *P*. *aeruginosa* strains damage and induce biofilm in other susceptible strains. Indeed, the link between pyocin-based cell damage and the biofilm response is consistent with experiments using clinical antibiotics, in which antibiotic resistance mutants require higher concentrations of antibiotics to elicit the biofilm response [10]. To confirm this effect in our assays, we tested a rifampicin resistant mutant of PAO1, with defined mutations in *rpoB* [55] and found that it does not respond to the same concentrations of rifampicin that trigger the biofilm responses in the wild type (S5 Fig). In sum, we see comparable effects on biofilm formation from diverse forms of cell damage. This suggests that cell damage from a diverse range of bacteria interactions, both between strains and between species, has the potential to promote biofilm formation.

### The Biofilm Response Is Observed in Microfluidic Experiments

**Fig 5:**
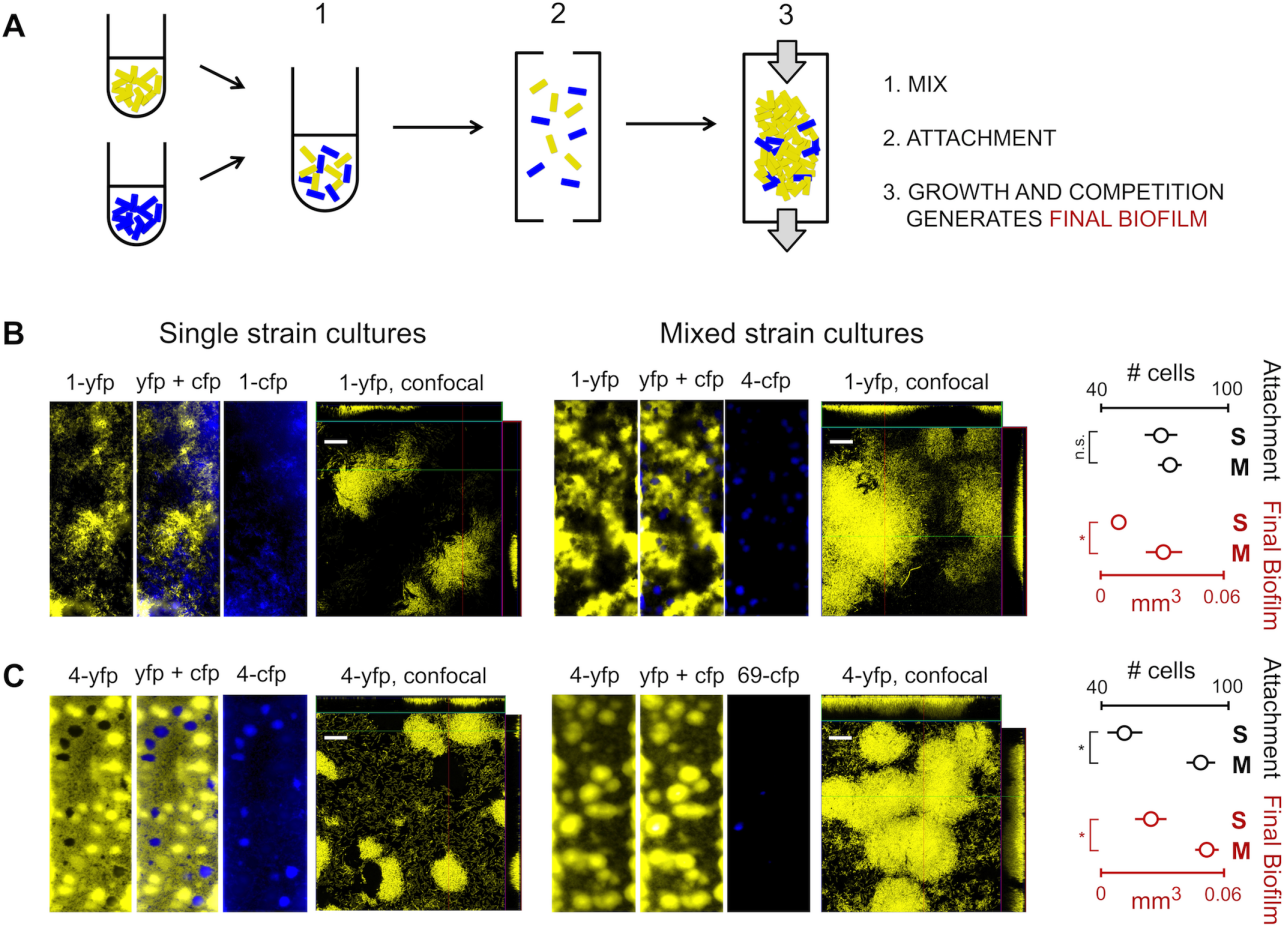
Strain mixing induces biofilm formation in *P*. *aeruginosa* in flow cells. A) Schematic of the experimental design, which involves mixing genotypes, initial attachment in the absence of flow, and finally, growth and competition under constant flow. Grey arrows represent the flow of nutrient media (tryptone broth). B) PAO1 biofilm compared to a mixed biofilm of PAO1 (strain 1) and strain 4. Within each image panel are three representative 10x epifluorescence micrographs of the flow cell channel showing the individual and merged YFP and CFP signals, and one 40x confocal image (scale bar is 30 μm) showing only the numerically-dominant *yfp* strain (autofluorescence in the CFP channel leads to a poor confocal signal). The charts at the far right show the initial attachment and final volume of biofilm of strain 1-*yfp* in single strain cultures (S) where 1-*yfp* is mixed with 1-*cfp* and in mixed strain cultures (M) where 1-*yfp* is mixed with 4-*cfp*. In both mixed and single cultures, the two differently-colored strains were initially mixed at a 1:1 ratio, *yfp*:*cfp*. Strain 1-*yfp* shows increased biofilm accumulation when mixed with 4, but cellular attachment is identical between single strain and mixed strain cultures. C) Strain 4 biofilm compared to a mixed biofilm of strain 4 and strain 69. In both mixed and single strain conditions, two differently-colored strains were initially mixed at a 3:1 ratio, *yfp*:*cfp*. Strain 4-*yfp* attaches more and makes more biofilm when mixed with 69. Error bars are 95% confidence intervals of the mean from six replicates, which combines data from two different days. *, *p* < 0.05 and n.s., not significant in a *t* test. Find numerical values in S1 Data.

Our experiments so far have utilized the crystal violet microtiter dish assay of standing cultures [48]. Although this experimental approach is widely used to quantify biofilm responses to antibiotics [13], it comes with two notable limitations. Firstly, indirect proxies are used to estimate biofilm formation (crystal violet staining) and planktonic cell numbers (optical density). Secondly, the presence of a large planktonic population in the standing culture means that the planktonic cells can affect the biofilm, and vice versa, in an undefined manner throughout the experiment. We therefore sought to recapitulate our results in a more defined assay using flow cells and direct imaging of the biofilms (Fig 5A).

We begin by studying one of our focal combinations, strain 1 (PAO1) and strain 4. As we find in the standing culture assay (Figs 1D and 2B), PAO1 dominates strain 4 and largely excludes it from the biofilm after 24 h (Fig 5B). Moreover, we observe an increase in the volume of biofilm formed by PAO1. Our observations in the less-defined crystal violet assays, therefore, are recapitulated in flow cell biofilms: strain-mixing results in a single strain dominating and an increase in biofilm formation. Do we also observe this biofilm response in strain 4, which was the focus of detailed analyses in the previous section? In order to study this, we needed to create a condition where strain 4 is not eliminated from the biofilm. Preliminary tests with our two focal mixtures (1 + 4, 4 + 69, Fig 1D) revealed that strain 4 will persist with strain 69, if it is over-represented at inoculation. We, therefore, tested strain 4 mixed with strain 69 in the flow cells at a 3:1 initial ratio. Under these conditions, strain 4 dominates the biofilms and largely excludes strain 69 and, most importantly, we see a clear increase in biofilm formation in strain 4 here when compared to its single-strain counterpart (Fig 5C).

Experiments using flow cells have a separate attachment phase at the beginning of the experiment to seed the surface with bacteria (see Methods). Counting attached cells after the attachment phase revealed that strain 4 attaches more in the presence of strain 69 but strain 1 does not attach more in the presence of strain 4 (Fig 5). Both initial attachment and subsequent biofilm accumulation, then, contribute to the response to strain mixing but while we always see increased biofilm formation, we do not always see an initial difference in attachment (also see next section). In sum, the flow cell experiments provide validation from a second assay that *P*. *aeruginosa* strains increase biofilm formation in response to the presence of competing strains.

### Antibiotic Gradients As Drivers of Biofilm Formation

Our data suggest that bacteriocins produced by natural isolates of *P*. *aeruginosa* act as a cue that drives biofilm formation. This raises the possibility that the widely reported biofilm responses to clinical antibiotics [10–17] also have their origins in responses to ecological competition from strains and species. But does the addition of antibiotics, or cell-free culture supernatant from competitors, also result in increased biofilm formation in a microfluidic device?

**Fig 6:**
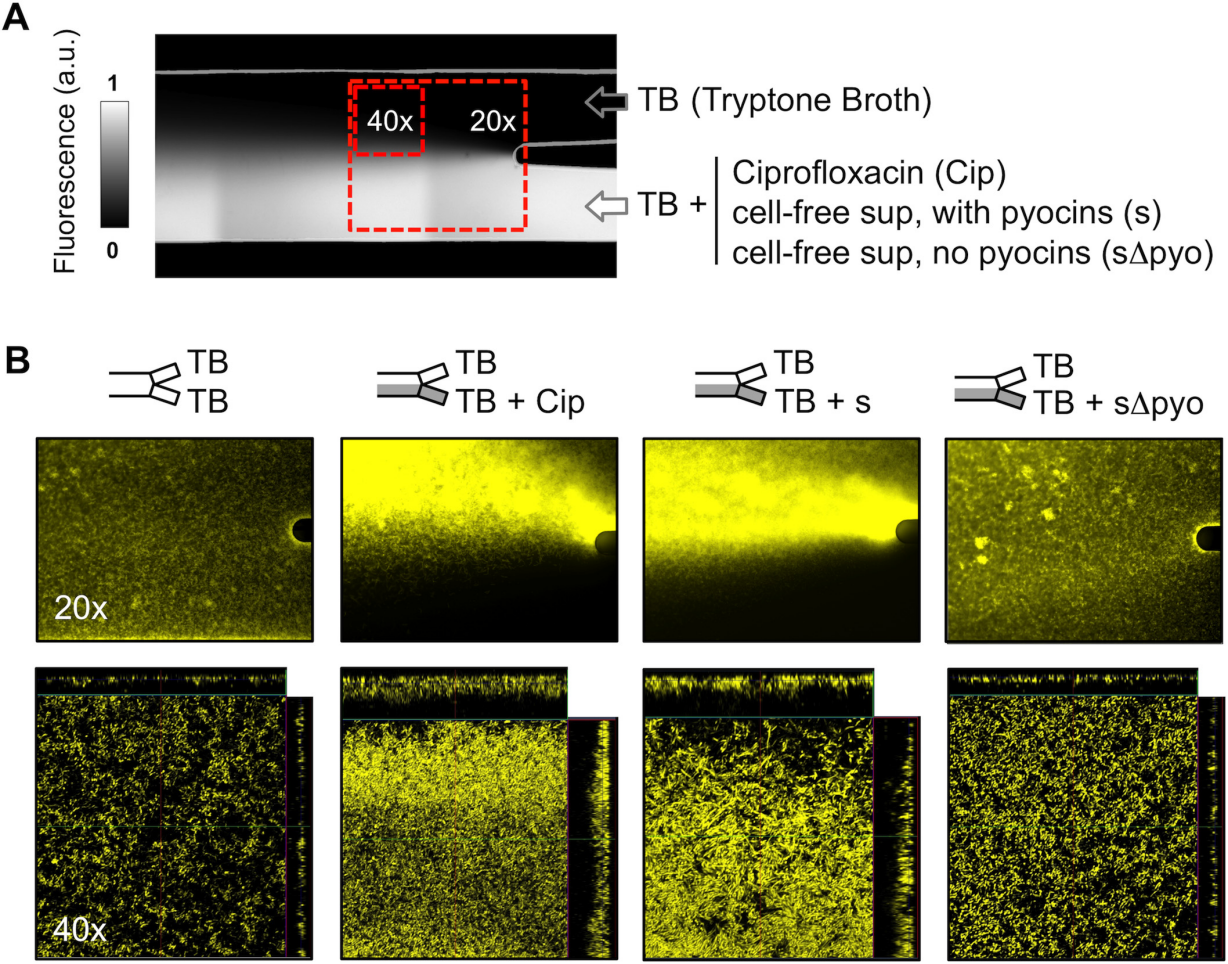
Antibiotic gradients drive biofilm formation. A) Image of the double inlet flow cell. Growth medium flows through both inlets but one also contains the test compound, which generates a gradient of the compound in the main chamber. Arrows indicate the direction of the flow. A fluorescent dye (fluorescein) has been added in this particular example (white) to illustrate the gradient created, but this is not present in the experiments in which we study the response of cells to antibiotics and cell-free supernatant (a.u., arbitrary units). B) Images corresponding to the red boxes shown in panel A, captured using a 20x objective (epifluorescence microscopy) and a 40x objective (confocal microscopy), respectively. 20x images are 350 μm by 450 μm, and 40x are 182 μm by 182 μm. Identical image collection and processing settings were used in all images to illustrate that ciprofloxacin (“Cip”) and cell-free culture supernatant from strain PA14 (“s”) both enhance the yellow signal when compared to controls at 20x magnification (“sΔpyo” is cell-free supernatant from the pyocin-null mutant and “TB” is tryptone broth, the standard medium). The confocal microscopy shows that the increase in the YFP signal in the epifluorescence images is explained by an increase in both cell density and volume of the biofilm.

Experiments with a single concentration of antibiotic revealed that the addition of cell-free culture supernatant or clinical antibiotics commonly results in cell detachment at the initial stage of the experiment. This is consistent with the effects of supernatants and antibiotics in the crystal violet assay being strongly concentration dependent, whereby lower concentrations cause the biofilm response and higher concentrations cause growth inhibition (Fig 3). We, therefore, developed a more robust method to study the effects of cell-free supernatant and antibiotics on biofilms in microfluidic devices. We moved to use microfluidic platforms with two inlet channels in which only one of the two inlets contains the antibiotics (Fig 6A).

The goal of this experimental approach was to create a gradient inside the main chamber with a wide range of antibiotic concentrations, from sub-lethal to lethal (see Methods). Our protocol always allows some parts of the chamber to contain negligible amounts of the antibiotic, which ensures that not all cells detach when antibiotics are flowed through. Importantly, the cells will experience a wide range of concentrations of antibiotic in a single experiment, and we can directly observe how they respond. Under these conditions, we observe a large increase in biofilm formation upon the addition of multiple classes of antibiotics in multiple strain backgrounds (Figs 6, S6 and S7). Our data show that antibiotics do indeed promote biofilm formation in flow cells. In addition, they support the value of studying gradients for understanding cellular responses to stress. Such gradients are common in biofilms [56,57], but their effects can be missed by static culture assays.

We next asked whether pyocins are also capable of promoting biofilm formation in our dual inlet experiment. In support of this, we find that flowing the cell-free culture supernatant of PA14 over strain 4 results in increased biofilm formation, but not when we use the supernatant of the pyocin null-mutant (Fig 6). What drives the increase in biofilm? Increased biofilm accumulation could occur through stronger attachment and better retention of cells throughout the experiment, or through the acceleration of cell division. In order to test the impact of pyocins on attachment, we allowed strain 4 cells to attach in the presence of cell free supernatant of PA14 and compared this to the effect of cell free supernatant of the PA14 pyocin null-mutant (S8 Fig). This shows that pyocins do indeed lead to increased attachment. This phenotype is not simply sedimentation of damaged cells. We flush unattached cells out before imaging and, importantly, we see increased attachment in the presence of pyocins both on the bottom and the top of the channel (S9 Fig). Moreover, propidium iodide staining shows that, concurrently, there is increased cell damage from the supernatant of PA14 than with the supernatant of the pyocin null mutant (S8 Fig), consistent with what we found for shaking culture (S3 Fig). We see, therefore, that pyocins from competitors both cause cell damage (S3 and S8 Figs) and an increase in biofilm formation (Figs 6, S4, S8 and S9).

The attachment experiments (S8 and S9 Figs) suggest that the biofilm response may occur via an increased propensity for cells to attach, and subsequently persist, in the biofilm. An increased rate of cell division could also contribute to the biofilm response in some or all strains. Direct assessments of cell division in three-dimensional biofilms is challenging and was not possible with our current methods. However, there is evidence from *Escherichia coli* that sub-lethal levels of antibiotics can increase growth rate [58]. More generally, there is evidence for widespread transcriptional and metabolic changes in response to antibiotics [16,59–66], which have recently been the focus of intense debate [62,67–70] but still lack evolutionary explanation. If these changes are also involved in the responses to other bacterial strains and species, it would suggest that many of the physiological and metabolic changes seen upon antibiotic use have their natural origins in competition sensing [41] and the ecology of the species concerned.

## Conclusions

Biofilms are central to microbiology. Despite this, we still understand relatively little about the drivers of biofilm formation in a natural context. Our data suggest that a major factor in biofilm formation in nature is ecological competition with other strains. We have shown that co-culturing strains of *P*. *aeruginosa* often leads to increased biofilm (Figs 1, 2 and 5). We have also shown that this increase in *P*. *aeruginosa* should not be interpreted as a cooperative interaction between strains. Mixing pairs of strains shows that one of the two strains is largely eliminated during the process of biofilm formation (Figs 2 and 5). Moreover, our data suggest that biofilm formation is stimulated as a response to cell damage caused by other strains: cell-free supernatant of one strain only induces biofilms in another strain when it can also causes cell damage (Figs 4, S3 and S8).

In pairwise mixtures of natural isolates, we have shown that one driver of the biofilm response are R and F pyocins, the narrow-spectrum antibiotics produced by *P*. *aeruginosa*. We, therefore, expect that pyocin-mediated competition will often be an inducer of biofilm formation in *P*. *aeruginosa*. However, our work also emphasizes that the biofilm responses to clinical antibiotics are highly comparable to those seen with pyocins. The different sets of antibiotics studied here have very different modes of action, including inhibition of DNA replication (ciprofloxacin), transcription (rifampicin), or translation (tetracycline) and, in the case of R and F pyocins, disruption of membrane potentials. The responses we observe, therefore, are very general and suggest that any toxin made by one *P*. *aeruginosa* strain has the potential to induce biofilm formation in another. Is there a common mechanism linking the diverse forms of cell damage to the biofilm response? Bacteria have a number of stress responses that are triggered by cell damage, which have been proposed as candidates for sensing the presence of competing strains [41]. Perhaps most notably, this includes the responses to oxidative stress, which is associated with the action of clinical antibiotics and diverse damaging agents from competing microbes [59,62,68,71,72].

What is the evolutionary basis of *P*. *aeruginosa* strains increasing biofilm formation in response to ecological competition? Mechanistically, our experiments suggest that the biofilm response is caused, at least in part, by an increase in surface-attachment in the presence of other strains. Whether a biofilm response is caused by differential attachment or another mechanism, the key effect is to increase the frequency of the responder relative to competing genotypes. We hypothesize that the evolutionary advantage of this increase in frequency is that it allows strains to better defend against and attack other genotypes. Biofilms are known to promote resistance to a wide range of stressors, including R pyocins [73]. In addition, competitive ability in a biofilm is expected to be linked to the relative density of each strain. At high density, cells can better control the environment with secreted products that favor their genotype over others [4,74]. Dominating a biofilm early, then, is likely to enable a genotype to dominate in the long term. In this model, the well-known biofilm responses to antibiotics [10–17] have their roots in natural selection to detect competing genotypes and respond in a manner that maximized the ability to defend and attack [39,41].

The fact that antibiotics are known to induce biofilm formation in diverse species suggests that ecological competition may increase biofilm formation in many microbial communities in which many strains and species meet. Consistent with this prediction, recent work on assemblage of multiple species, including known antibiotic producers, found that strain mixing increases biofilm formation [26,30,31], although it was interpreted there as cooperation between species. Understanding the effects of ecological competition on biofilm formation also has implications for health and disease. An important new idea in the treatment of disease is that we can harness ecological competition between microbes to our advantage [75,76]. Probiotic supplements for the gut are already an important industry [77], and a major goal of synthetic biology is to arm benign bacteria with the antibiotics they need to combat disease-causing strains [78]. We must be cautious, then, that this escalation of ecological competition does not result in the outcomes that we observe here: a more abundant pathogen.

## Methods

### Strains

A total of 22 strains of *Pseudomonas aeruginosa* were used, including both clinical and environmental isolates from the Kolter strain collection (S2 Table). A subset of these strains was subject to detailed analyses, including the well-studied clinical strain PAO1 (here referred to as strain 1), and strains 4, 7, 19, and 69. These five strains were chromosomally tagged with constitutively-expressed fluorescent tags (*cfp* or *yfp*) using the mini-*Tn*7 system [50]. The pyocin-null *prtR* ^*S153A*^ (constructed as in [52]), producing constitutively active repressor, and its parental strain PA14 were kindly provided by Jon Penterman. The R and F pyocin mutants are from the PA14 Non-Redundant Transposon Insertion Mutant Set [79]. We found no differences in our assays between the two sources of PA14 strains and only show the data from the Penterman source in the figures. Other strains are described in S3 Table.

### Static Biofilm Assays

These assays were based on standard protocols [48]. Briefly, cells from overnight cultures of *P*. *aeruginosa* were sub-cultured and from these, exponential phase cells were inoculated in peg-lidded 96-well plates (Nunc, conical bottom) in 100 μl tryptone broth (10 g Bacto triptone per 1 l water) at starting optical density of 0.0025 (A_600_), and allowed to grow at 22°C. After 24 h, 50 μl of medium was removed and the optical density at A_600_ was measured to estimate the density of planktonic cells. Pegs were then washed, stained with 0.1% crystal violet, washed again, and then the remaining stain was dissolved with 200 μl of 33% acetic acid. Finally, the optical density at A_595_ was measured and used as a proxy for biofilm formation. For direct strain-mixing experiments (Figs 1C, 1D and 2), we used plates without pegs because this allows cells to be more easily removed and sampled from the biofilms when required (see below). The only substantive difference is that the biofilm on the edge of the well is assessed using crystal violet rather than the biofilm on a peg. We confirmed that the two assays give very similar results (S10 Fig). While the experiments presented in the main text were done in tryptone broth, the biofilm response was also investigated in other standard bacterial growth media, including Luria broth (LB), *Pseudomonas* minimal medium (PMM) and casamino acid (CAA) medium, along with different concentrations of iron or buffer, which did not have qualitative effects in the cellular responses to mixing (S1 Fig).

### Antibiotics

Three antibiotics belonging to different structural families, namely ciprofloxacin, rifampicin, and tetracycline, were chosen to recapitulate the effect of sub-inhibitory concentrations of antibiotics on biofilm formation of *P*. *aeruginosa* [13]. Stock solutions of each antibiotic (obtained from Sigma Chemical Company) were stored at -20°C and diluted to the desired concentration on the day of each experiment. All antibiotics experiments used tryptone broth as the growth medium. Biofilm formation and growth in shaking culture were assayed as detailed in the sections “Static Biofilm Assays” (above) and “Growth Curves” (below), respectively. For the dual-flow microfluidic experiments (Fig 6) we used 10x the concentration that inhibited growth in shaking culture, as shown in Fig 1. These are 1 μg/mL for ciprofloxacin, 50 μg/mL for rifampicin, and 1,000 μg/mL for tetracycline.

### Strain Mixing Experiments

The initial survey of how strain mixing affects biofilm formation was based on 22 strains of *P*. *aeruginosa*. Biofilm formation after 24 h was evaluated in 22 single strains, 22 pair-wise combinations, eight combinations of five strains, and two of ten strains. Strains were evenly mixed based upon the optical density of stationary phase cultures. The strain combinations in the mixtures were chosen to ensure a near-even representation of the strains across the mixtures. Each strain occurred twice in the pairs, twice in the mixtures of fives (with one strain appearing only once), and once in a mixture of ten (with one strain excluded). Sixteen replicates were performed in each experiment for each strain or mixture, to give a total of 432 biofilm assays in each experiment. All treatments were performed on two different days.

The second set of experiments mixed five fluorescently-labeled strains in all combinations. We assessed biofilm formation and planktonic cell density in 96 well plates after 24 h. Samples of planktonic cells and biofilm cells were taken from each treatment to record the frequency of the two strains using fluorescence microscopy. The biofilm sample was taken by vigorously shaking the washed plates in phosphate buffer for one hour (experiments revealed that this treatment gives identical frequency estimates as shaking with 500 μm glass beads and removing the whole biofilm). In addition, direct images of the biofilms were taken by growing strains in 2 ml of media containing 25 x 25 mm^2^ glass coverslips in 6-well plates under conditions otherwise identical to the 96-well plate assay. The bacteria form a biofilm on the coverslip slightly below the air-water interface, which is then visualized by fluorescent microscopy (Fig 2).

All experiments were repeated twice on different days. Comparison of these data with the pair-wise mixtures in the first experiments (above) confirmed that the mixing responses were not affected by fluorescent labeling. All methods were the same as before with the addition that we diluted overnight cultures of each strain and grew them for 2 h to obtain exponential phase cells to inoculate the biofilm assays (Fig 2). This did not affect biofilm formation, but it helped reduce variability in the strain frequency in the mixtures. For the two large-scale strain-mixing experiments (Figs 1C and 2), we present data that is the combination of the two repeats from different days as variability between days was relatively high.

### Microfluidic Biofilm Assays

Biofilm formation was also monitored under laminar flow conditions using the BioFlux 200 system (Fluxion Biosciences, South San Francisco, CA, United States). Experiments were performed essentially as described elsewhere [80]. First, microfluidic channels (350 μm x 75 μm x 4.89 mm for width, height, and length, respectively) were primed with growth media, here tryptone broth, to prevent air bubbles from entering the channels on subsequent loading steps, and just after, inoculated with cells at the exponential phase. The concentration of these cultures was adjusted so that the optical density at 600 nm in tryptone broth was 0.25. Then, after the attachment period without flow (20 min at 22°C), fresh medium was pumped from inlet wells through the channels to outlet, firstly with a flow rate of 40 μl/h for 20 min to flush out planktonic cells, and then with a constant flow rate of 4 μl/h until the end of the experiments (24 or 48 h). To address the effect of pyocins on cellular attachment we allowed cells to attach in the channels for 20 min with tryptone broth made with 10% cell-free supernatant, either from strain PA14 WT or a mutant that has the repressor of pyocins, *prtR*, mutated to a version that is constitutively active (S3 Table). We then flushed out planktonic cells, as explained above, and imaged to assess attachment.

For consistency, we used only one plate type for all microfluidic experiments, the “Invasion” plate of Fluxion Biosciences that has two parallel inlets and one outlet (Fig 6). When studying co-cultures, the two inlets contained the same medium, and when studying the effect of antibiotic gradients we added the required concentration of the antibiotic (or cell-free culture supernatant) to one of the inlets only, thus creating a gradient of the compound being tested in the main chamber. Each experiment was repeated twice, on different days, and the images of biofilms taken come from the middle of the channels, where the channels are the flattest, when accessing the effects of strain mixing. In all treatments, images were taken at the same distance from the respective inlets. When accessing the effect of antibiotics or cell-free supernatant we imaged directly downstream of the inlets, as shown in Fig 6, where the gradient is the steepest.

### Study of Diffusible Substances

To access the effects of diffusible substances in the biofilm response, two sets of experiments were performed. Firstly, we used 96-well Transwell plates (Corning) with two different inlays separated by a 0.4 μm pore size membrane. In these experiments, the strain in the bottom of the well was inoculated at an optical density of 0.0025 (A_600_) in 75 μl of media. This bottom strain was the tester strain in which biofilm formation was analyzed. The top strain was the inducer and was inoculated at a higher density of 0.1 (A_600_) with 50 μl media. These revealed cell-to-cell contact is not strictly necessary for the biofilm response (Fig 3A). To investigate this further, we studied responses of strains to cell-free supernatants from other strains. The supernatant of the strains were obtained as follows. Exponential phase cells of all strains were inoculated in 6-well plates in 4 ml of tryptone broth at starting optical density of 0.025 (A_600_), and allowed to grow at 22°C for 21 h. Cultures were then spun down (3,000 g during 10 min) and filtrated using 0.22 μm filters (Millipore). The spent media assay followed the same methods for the biofilm assay, except that exponential phase cells of the responder strain were inoculated in media containing (2.5/25%) cell-free culture supernatant from itself or others.

Fractionation of the cell free supernatant was performed by GE 1 ml resource Q anion exchange chromatography (buffer A: 50 mM Tris-HCl [pH 7.5], 2% glycerol, 2 mM β-mercaptoethanol; buffer B: buffer A + 1 M NaCl) followed by GE Superdex 200 10/300 GL gel filtration in SEC buffer. The active fraction, as determined by the crystal violet assay (Fig 4), was subject to mass spectrometry by the FAS Center for Systems Biology Core Facilities, Harvard University, US. Briefly, the sample was reduced, carboxyamidomethylated and digested with trypsin. Peptide sequence analysis of each digestion mixture was then performed by microcapillary reversed phase high-performance liquid chromatography coupled with nanoelectrospray tandem mass spectrometry (μLC-MS/MS) on an LTQ-Orbitrap mass spectrometer (ThermoFisher Scientific, San Jose, CA, US).

### Growth Curves

Exponential phase cells from overnight cultures were inoculated in flat-bottomed 96-well plates (Nunc) in 200 μl of tryptone broth at the starting optical density (A_600_) of 0.0025, as for the biofilm assays, and allowed to grow at 22°C, shaking, inside a plate reader (Tecan, Infinite M200 Pro). Test treatments were supplemented with 25% of cell-free supernatant from PA14, the respective pyocin-null, or distilled water (control). The optical density at A_600_ was measured automatically every 500 s. When required, cell damage was assessed by staining shaking cultures or attached cells in a microfluidic device with propidium iodide (at the final concentration of 3 μl/ml)[81]. Propidium iodide-stained cells were visualized using epifluorescence microscopy with DsRed-based filters.

### Fluorescence and Confocal Microscopy

Images of BioFlux biofilms were obtained using a Zeiss confocal laser scanning microscope (LSM 700), a digital camera (AxioCam MRm), and the associated Zen 2011 software (blue or black edition, for fluorescence or confocal microscopy, respectively). Objectives used were 10x, 20x, or 40x, as specified in the figure legends.

### Image Analysis

A MATLAB (MathWorks) script was used to determine the number of attached cells on the surface of microfluidics, along with the volume of biofilms after 24 h and 48 h of incubation. The volume calculation uses the area of fluorescence signal of biofilms in each confocal slice, which is then summed across the slices in *z*-plane.

## Supporting Information

S1 Data Excel file containing the data for Figs 1A–1D, 2A, 2B, 3A, 3B, 4A–4D, 5B–5C, S1, S2, S3, S4, S5 and S10. (XLSX)

S1 Fig The biofilm response is observed across multiple environmental conditions. Biofilm formation in single-strain and mixed-strain cultures is shown across a range of conditions. We compared biofilm formation across (i) three nutrient concentrations with casamino acids (CAA) media: 0.1% CAA (low), 0.5% CAA (medium), 2.5% CAA (high); (ii) three iron levels with casamino acids media (100 mg apotransferin + 1 μM FeCl, 100mg apotransferin + 5 μM FeCl, no added apotransferin or FeCl); (iii) three buffering levels with casamino acids media (Unbuffered, 25 mM HEPES, 75mM HEPES); and (iv) four standard media including Luria broth (LB), tryptone broth (TB), *Pseudomonas* minimal medium (PMM), and casamino acid (CAA) medium. Note that the CAA standard media treatment is replotted in each quadrant for comparison, where it represents medium nutrients, unlimited iron, no buffering, and CAA, respectively. In most cases, strain 4 is outcompeted and represents <5% of the planktonic cells after 24 h. A few exceptions are marked with an arrow where strain 4 is >5% frequency in the planktonic cells. Most notably, the 4 + 69 mixtures marked with an arrow have strain 4 at >90% of planktonic cells, showing how the outcome of competition is strongly influenced by the environment, while the biofilm response is much less so. Error bars indicate the 95% confidence intervals of the mean. Find numerical values in S1 Data.
(TIFF)

S2 Fig Dose-dependent effect of strain mixing on the biofilm response of strains 4 and PA14. Error bars are 95% confidence intervals of the mean. Find numerical values in S1 Data.
(TIFF)

S3 Fig Pyocin-induced cell damage in shaking culture. Cell-free supernatant from wild-type (WT) PA14 inhibits the growth of strain 4, as shown in Fig 3. The observed growth lag (left-hand plots) is driven by pyocins, the narrow spectrum antibiotics of *P*. *aeruginosa*, and is associated with cell damage (right hand images). After 5 h of growth in the presence of 25% of PA14 cell free supernatant with pyocins (“s14”) or without (“s14Δpyo”) we stained cultures of strain 4 with propidium iodide, and imaged cells using epifluorescence microscopy (see S3 Table for details of PA14 strains). The responder genotype (strain 4) is tagged with a fluorescent protein (YFP), and damaged cells are seen in red. The growth curves on the left are means of eight independent replicates and images on the right are representative images of cell damage in the cultures at the end of the growth period (large red blob is an out-of-focus damaged cell). Images from each fluorescent channel were collected and processed using the same settings to ensure consistency. Scale bar is 20 μm. Find numerical values in S1 Data.
(TIFF)

S4 Fig Pyocin-induced biofilm formation in additional strains of *P*. *aeruginosa*. Shown are the effects of cell-free culture supernatants from PA14 and the pyocin-null mutant (14Δpyo) on two natural isolates from Fig 1C. Pyocin-induced biofilm formation is also observed in PAK, a strain known to be susceptible to pyocins from PA14. Error bars are 95% confidence intervals of the mean. Find numerical values in S1 Data.
(TIFF)

S5 Fig Effect of antibiotic resistance on antibiotic-induced biofilm formation. Strain PAO1 exhibits a rifampicin-induced biofilm response, but its rifampicin resistant mutant does not. Error bars are 95% confidence intervals of the mean. Find numerical values in S1 Data.
(TIFF)

S6 Fig Different classes of clinical antibiotics promote biofilm formation in flow cells. Gradients of rifampicin (Rif) and tetracycline (Tet) drive biofilm accumulation in strain 4 as one finds with gradients of ciprofloxacin (Fig 6). The effect is particularly clear after 48 h of incubation when the biofilm increase is such that blocks the flow. For each treatment we show representative epifluorence microscopy images captured with 10x lens (scale bar is 100 μm for “Brightfield,” “YFP,” and “Merged”), as well as representative confocal images captured with 40x lens (scale bar is 30 μm). Epifluorence images were collected and processed using the same settings to ensure consistency. Confocal images show that the increase in the YFP signal with antibiotic gradients is explained by an increase in both cell density and volume of the biofilm.
(TIFF)

S7 Fig Antibiotics promote biofilm formation in flow cells in a second strain background (strain 69). Gradients of ciprofloxacin (Cip), rifampicin (Rif), and tetracycline (Tet) drive biofilm accumulation. The effect is particularly clear after 48 h of incubation when the biofilm increase is such that it can block the flow. For each treatment we show representative epifluorence microscopy images captured with 10x lens (scale bar, 100 μm for “Brightfield,” “YFP,” and “Merged”), as well as representative confocal images captured with 40x lens (scale bar is 30 μm). Confocal images show that the increase in the YFP signal with antibiotic gradients is explained by an increase in both cell density and volume of the biofilm.
(TIFF)

S8 Fig Pyocins from competitor genotypes drive both attachment and cell damage in *P*. *aeruginosa*. Cell-free supernatant from WT PA14 (s14) promotes surface attachment of strain 4 when compared to supernatant from the respective pyocin-null genotype (s14Δpyo) as observed by two representative regions of each channel. Following the attachment phase we stained *yfp*-labeled cells with propidium iodide to test for cell damage (red). Images from each fluorescent channel were collected and processed using the same settings to ensure consistency. Scale bar is 20 μm. Experiments were repeated on different days to validate results.
(TIFF)

S9 Fig Pyocin-induced attachment occurs on the top and bottom of the microfluidic device. Specifically, we see increased cellular attachment of YFP-labeled strain 4 in the presence of pyocin-containing cell-free supernatant from WT PA14 (s14) on both the bottom (glass) and top (PDMS) of channels, when compared to supernatant from the respective pyocin-null genotype (s14Δpyo). *p* < 0.05 in unpaired *t* tests at 5% significance. We show two representative images of each condition. Scale bar is 20 μm.
(TIFF)

S10 Fig Comparison of standard assays (A) and peg assays (B) on mixing-induced biofilm formation. In standard assays, the biofilms measured are those attached to the edge of the wells in microtiter plates. In peg assays, the biofilms measured are those attached to pegs inserted into the wells. Here we show dose-dependent effects of strain mixing on the biofilm response of strains 1 and 4 (left) and 4 and 69 (right). Increased biofilm due to strain mixing is seen in both assays. Error bars are 95% confidence intervals of the mean. Find numerical values in S1 Data.
(TIFF)

S1 Table Tryptic peptide fragments identified by mass spectrometry found by Protein Mass Spectrometry with homology to pyocin proteins. (TIFF)

S2 Table Natural isolates of *Pseudomonas aeruginosa* used in this study. (TIFF)

S3 Table Additional strains used in this study. (TIFF)

## Abbreviations:

CAA: casamino acid
CFP: cyan fluorescent protein
LB: Luria broth
PMM: Pseudomonas minimal medium
TB: tryptone broth
YFP: yellow fluorescent protein
WT: wild-type

## References

[1] Costerton JW (1995) Overview of microbial biofilms. J Ind Microbiol 15: 137–140. 8519468 10.1007/BF01569816

[2] Kolter R, Greenberg EP (2006) Microbial sciences—The superficial life of microbes. Nature 441: 300–302. 16710410 10.1038/441300a

[3] Monds RD, O'Toole GA (2009) The developmental model of microbial biofilms: ten years of a paradigm up for review. Trends in Microbiology 17: 73–87. 10.1016/j.tim.2008.11.001 19162483

[4] Nadell CD, Xavier JB, Foster KR (2009) The sociobiology of biofilms. FEMS Microbiol Rev 33: 206–224. 10.1111/j.1574-6976.2008.00150.x 19067751

[5] Hall-Stoodley L, Costerton JW, Stoodley P (2004) Bacterial biofilms: From the natural environment to infectious diseases. Nature Reviews Microbiology 2: 95–108. 15040259 10.1038/nrmicro821

[6] Lopez D, Vlamakis H, Kolter R (2010) Biofilms. Cold Spring Harbor Perspectives in Biology 2: a000398 10.1101/cshperspect.a000398 20519345 PMC2890205

[7] Romling U, Kjelleberg S, Normark S, Nyman L, Uhlin BE, et al (2014) Microbial biofilm formation: a need to act. J Intern Med 276: 98–110. 10.1111/joim.12242 24796496

[8] Beloin C, Ghigo JM (2005) Finding gene-expression patterns in bacterial biofilms. Trends in Microbiology 13: 16–19. 15639627 10.1016/j.tim.2004.11.008

[9] Karatan E, Watnick P (2009) Signals, Regulatory Networks, and Materials That Build and Break Bacterial Biofilms. Microbiology and Molecular Biology Reviews 73: 310–347. 10.1128/MMBR.00041-08 19487730 PMC2698413

[10] Elliott D, Burns JL, Hoffman LR (2010) Exploratory Study of the Prevalence and Clinical Significance of Tobramycin-Mediated Biofilm Induction in *Pseudomonas aeruginosa* Isolates from Cystic Fibrosis Patients. Antimicrobial Agents and Chemotherapy 54: 3024–3026. 10.1128/AAC.00102-10 20404125 PMC2897298

[11] Haddadin RNS, Saleh S, Al-Adham ISI, Buultjens TEJ, Collier PJ (2010) The effect of subminimal inhibitory concentrations of antibiotics on virulence factors expressed by *Staphylococcus aureus* biofilms. Journal of Applied Microbiology 108: 1281–1291. 10.1111/j.1365-2672.2009.04529.x 19778348

[12] Hoffman LR, D'Argenio DA, MacCoss MJ, Zhang ZY, Jones RA, et al (2005) Aminoglycoside antibiotics induce bacterial biofilm formation. Nature 436: 1171–1175. 16121184 10.1038/nature03912

[13] Kaplan JB (2011) Antibiotic-induced biofilm formation. International Journal of Artificial Organs 34: 737–751. 10.5301/ijao.5000027 22094552

[14] Kaplan JB, Izano EA, Gopal P, Karwacki MT, Kim S, et al (2012) Low levels of beta-lactam antibiotics induce extracellular DNA release and biofilm formation in *Staphylococcus aureus* . MBio 3: e00198–00112. 10.1128/mBio.00198-12 22851659 PMC3419523

[15] Kumar A, Ting YP (2013) Effect of sub-inhibitory antibacterial stress on bacterial surface properties and biofilm formation. Colloids and Surfaces B-Biointerfaces 111: 747–754. 10.1016/j.colsurfb.2013.07.011 23934235

[16] Linares JF, Gustafsson I, Baquero F, Martinez JL (2006) Antibiotics as intermicrobial signaling agents instead of weapons. Proceedings of the National Academy of Sciences of the United States of America 103: 19484–19489. 17148599 10.1073/pnas.0608949103 PMC1682013

[17] Wang Q, Sun FJ, Liu Y, Xiong LR, Xie LL, et al (2010) Enhancement of Biofilm Formation by Subinhibitory Concentrations of Macrolides in icaADBC-Positive and-Negative Clinical Isolates of *Staphylococcus epidermidis* . Antimicrobial Agents and Chemotherapy 54: 2707–2711. 10.1128/AAC.01565-09 20231401 PMC2876384

[18] Andersson DI, Hughes D (2014) Microbiological effects of sublethal levels of antibiotics. Nat Rev Microbiol 12: 465–478. 10.1038/nrmicro3270 24861036

[19] Davies J, Spiegelman GB, Yim G (2006) The world of subinhibitory antibiotic concentrations. Curr Opin Microbiol 9: 445–453. 16942902 10.1016/j.mib.2006.08.006

[20] Fajardo A, Martinez JL (2008) Antibiotics as signals that trigger specific bacterial responses. Current Opinion in Microbiology 11: 161–167. 10.1016/j.mib.2008.02.006 18373943

[21] Yim G, Wang HMH, Davies J (2007) Antibiotics as signalling molecules. Philosophical Transactions of the Royal Society B-Biological Sciences 362: 1195–1200. 10.1098/rstb.2007.2044 PMC2435582 17360275

[22] Andersson S, Rajarao GK, Land CJ, Dalhammar G (2008) Biofilm formation and interactions of bacterial strains found in wastewater treatment systems. Fems Microbiology Letters 283: 83–90. 10.1111/j.1574-6968.2008.01149.x 18422628

[23] Burmolle M, Ren DW, Bjarnsholt T, Sorensen SJ (2014) Interactions in multispecies biofilms: do they actually matter? Trends in Microbiology 22: 84–91. 10.1016/j.tim.2013.12.004 24440178

[24] Burmolle M, Webb JS, Rao D, Hansen LH, Sorensen SJ, et al (2006) Enhanced biofilm formation and increased resistance to antimicrobial agents and bacterial invasion are caused by synergistic interactions in multispecies biofilms. Appl Environ Microbiol 72: 3916–3923. 16751497 10.1128/AEM.03022-05 PMC1489630

[25] Elias S, Banin E (2012) Multi-species biofilms: living with friendly neighbors. FEMS Microbiol Rev 73: 310–347. 10.1111/j.1574-6976.2012.00325.x 22229800

[26] Lee KWK, Periasamy S, Mukherjee M, Xie C, Kjelleberg S, et al (2014) Biofilm development and enhanced stress resistance of a model, mixed-species community biofilm. Isme Journal 8: 894–907. 10.1038/ismej.2013.194 24152718 PMC3960537

[27] Little AEF, Robinson CJ, Peterson SB, Raffa KE, Handelsman J (2008) Rules of Engagement: Interspecies Interactions that Regulate Microbial Communities. Annual Review of Microbiology 62: 375–401. 10.1146/annurev.micro.030608.101423 18544040

[28] Moller S, Sternberg C, Andersen JB, Christensen BB, Ramos JL, et al (1998) In situ gene expression in mixed-culture biofilms: Evidence of metabolic interactions between community members. Applied and Environmental Microbiology 64: 721–732. 9464414 10.1128/aem.64.2.721-732.1998 PMC106108

[29] Pande S, Merker H, Bohl K, Reichelt M, Schuster S, et al (2013) Fitness and stability of obligate cross-feeding interactions that emerge upon gene loss in bacteria. ISME J 8: 953–962. 10.1038/ismej.2013.211 24285359 PMC3996690

[30] Ren D, Madsen JS, de la Cruz-Perera CI, Bergmark L, Sorensen SJ, et al (2014) High-Throughput Screening of Multispecies Biofilm Formation and Quantitative PCR-Based Assessment of Individual Species Proportions, Useful for Exploring Interspecific Bacterial Interactions. Microb Ecol 68: 146–154. 10.1007/s00248-013-0315-z 24337804

[31] Ren D, Madsen JS, Sorensen SJ, Burmolle M (2014) High prevalence of biofilm synergy among bacterial soil isolates in cocultures indicates bacterial interspecific cooperation. ISME J 9: 81–89. 10.1038/ismej.2014.96 24936766 PMC4274433

[32] Chandler JR, Heilmann S, Mittler JE, Greenberg EP (2012) Acyl-homoserine lactone-dependent eavesdropping promotes competition in a laboratory co-culture model. Isme Journal 6: 2219–2228. 10.1038/ismej.2012.69 22763647 PMC3504959

[33] Drescher K, Nadell CD, Stone HA, Wingreen NS, Bassler BL (2014) Solutions to the Public Goods Dilemma in Bacterial Biofilms. Current Biology 24: 50–55. 10.1016/j.cub.2013.10.030 24332540 PMC3935403

[34] Mitri S, Foster KR (2013) The genotypic view of social interactions in microbial communities. Annu Rev Genet 47: 247–273. 10.1146/annurev-genet-111212-133307 24016192

[35] Nadell CD, Bassler BL (2011) A fitness trade-off between local competition and dispersal in *Vibrio cholerae* biofilms. Proceedings of the National Academy of Sciences of the United States of America 108: 14181–14185. 10.1073/pnas.1111147108 21825170 PMC3161532

[36] Foster KR, Bell T (2012) Competition, not cooperation, dominates interactions among culturable microbial species. Curr Biol 22: 1845–1850. 10.1016/j.cub.2012.08.005 22959348

[37] Hibbing ME, Fuqua C, Parsek MR, Peterson SB (2010) Bacterial competition: surviving and thriving in the microbial jungle. Nature Reviews Microbiology 8: 15–25. 10.1038/nrmicro2259 19946288 PMC2879262

[38] Oliveira NM, Niehus R, Foster KR (2014) Evolutionary limits to cooperation in microbial communities. Proceedings of the National Academy of Sciences of the United States of America 111: 17941–17946. 10.1073/pnas.1412673111 25453102 PMC4273359

[39] Ratcliff WC, Denison RF (2011) Microbiology. Alternative actions for antibiotics. Science 332: 547–548. 10.1126/science.1205970 21527704

[40] Bernier SP, Surette MG (2013) Concentration-dependent activity of antibiotics in natural environments. Front Microbiol 4: 20 10.3389/fmicb.2013.00020 23422936 PMC3574975

[41] Cornforth DM, Foster KR (2013) Competition sensing: the social side of bacterial stress responses. Nature Reviews Microbiology 11: 285–293. 10.1038/nrmicro2977 23456045

[42] Keller L, Surette MG (2006) Communication in bacteria: an ecological and evolutionary perspective. Nature Reviews Microbiology 4: 249–258. 16501584 10.1038/nrmicro1383

[43] Maynard Smith J, Harper D (2003) Animal signals Oxford: Oxford University Press ix, 166 p. p.

[44] Darwin C (1859) On the origin of species by means of natural selection. London,: J. Murray. ix, 1, 502 p. p.

[45] Burns JL, Gibson RL, McNamara S, Yim D, Emerson J, et al (2001) Longitudinal assessment of *Pseudomonas aeruginos*a in young children with cystic fibrosis. J Infect Dis 183: 444–452. 11133376 10.1086/318075

[46] Mahenthiralingam E, Campbell ME, Foster J, Lam JS, Speert DP (1996) Random amplified polymorphic DNA typing of *Pseudomonas aeruginosa* isolates recovered from patients with cystic fibrosis. J Clin Microbiol 34: 1129–1135. 8727889 10.1128/jcm.34.5.1129-1135.1996 PMC228968

[47] Renders N, Verbrugh H, Van Belkum A (2001) Dynamics of bacterial colonisation in the respiratory tract of patients with cystic fibrosis. Infect Genet Evol 1: 29–39. 12798048 10.1016/s1567-1348(01)00004-1

[48] O'Toole GA, Kolter R (1998) Flagellar and twitching motility are necessary for *Pseudomonas aeruginosa* biofilm development. Molecular Microbiology 30: 295–304. 9791175 10.1046/j.1365-2958.1998.01062.x

[49] Savoia D, Zucca M (2007) Clinical and environmental Burkholderia strains: Biofilm production and intracellular survival. Current Microbiology 54: 440–444. 17457645 10.1007/s00284-006-0601-9

[50] Lambertsen L, Sternberg C, Molin S (2004) Mini-Tn7 transposons for site-specific tagging of bacteria with fluorescent proteins. Environ Microbiol 6: 726–732. 15186351 10.1111/j.1462-2920.2004.00605.x

[51] Michel-Briand Y, Baysse C (2002) The pyocins of *Pseudomonas aeruginosa* . Biochimie 84: 499–510. 12423794 10.1016/s0300-9084(02)01422-0

[52] Penterman J, Singh PK, Walker GC (2014) Biological Cost of Pyocin Production during the SOS Response in *Pseudomonas aeruginosa* . Journal of Bacteriology 196: 3351–3359. 10.1128/JB.01889-14 25022851 PMC4135695

[53] Heo YJ, Chung IY, Choi KB, Cho YH (2007) R-Type pyocin is required for competitive growth advantage between *Pseudomonas aeruginosa* strains. Journal of Microbiology and Biotechnology 17: 180–185. 18051371

[54] Kohler T, Donner V, van Delden C (2010) Lipopolysaccharide as Shield and Receptor for R-Pyocin-Mediated Killing in *Pseudomonas aeruginosa* . Journal of Bacteriology 192: 1921–1928. 10.1128/JB.01459-09 20118263 PMC2838038

[55] Qi Q, Preston GM, MacLean RC (2014) Linking System-Wide Impacts of RNA Polymerase Mutations to the Fitness Cost of Rifampin Resistance in *Pseudomonas aeruginosa* . Mbio 5: e01562 10.1128/mBio.01562-14 25491352 PMC4324240

[56] Tseng BS, Zhang W, Harrison JJ, Quach TP, Song JL, et al (2013) The extracellular matrix protects *Pseudomonas aeruginosa* biofilms by limiting the penetration of tobramycin. Environmental Microbiology 15: 2865–2878. 10.1111/1462-2920.12155 23751003 PMC4045617

[57] Stewart PS, Franklin MJ (2008) Physiological heterogeneity in biofilms. Nature Reviews Microbiology 6: 199–210. 10.1038/nrmicro1838 18264116

[58] Migliore L, Rotini A, Thaller MC (2013) Low Doses of Tetracycline Trigger the *E*. *Coli* Growth: A Case of Hormetic Response. Dose-Response 11: 550–557. 10.2203/dose-response.13-002.Migliore 24298230 PMC3834746

[59] Dwyer DJ, Belenky PA, Yang JH, MacDonald IC, Martell JD, et al (2014) Antibiotics induce redox-related physiological alterations as part of their lethality. Proc Natl Acad Sci U S A 111: E2100–2109. 10.1073/pnas.1401876111 24803433 PMC4034191

[60] Dwyer DJ, Kohanski MA, Hayete B, Collins JJ (2007) Gyrase inhibitors induce an oxidative damage cellular death pathway in *Escherichia coli* . Molecular Systems Biology 3: 91 17353933 10.1038/msb4100135 PMC1847949

[61] Goh EB, Yim G, Tsui W, McClure J, Surette MG, et al (2002) Transcriptional modulation of bacterial gene expression by subinhibitory concentrations of antibiotics. Proceedings of the National Academy of Sciences of the United States of America 99: 17025–17030. 12482953 10.1073/pnas.252607699 PMC139263

[62] Kohanski MA, Dwyer DJ, Collins JJ (2010) How antibiotics kill bacteria: from targets to networks. Nature Reviews Microbiology 8: 423–435. 10.1038/nrmicro2333 20440275 PMC2896384

[63] Kohanski MA, Dwyer DJ, Hayete B, Lawrence CA, Collins JJ (2007) A common mechanism of cellular death induced by bactericidal antibiotics. Cell 130: 797–810. 17803904 10.1016/j.cell.2007.06.049

[64] Kohanski MA, Dwyer DJ, Wierzbowski J, Cottarel G, Collins JJ (2008) Mistranslation of Membrane Proteins and Two-Component System Activation Trigger Antibiotic-Mediated Cell Death. Cell 135: 679–690. 10.1016/j.cell.2008.09.038 19013277 PMC2684502

[65] Tsui WHW, Yim G, Wang HHM, McClure JE, Surette MG, et al (2004) Dual effects of MLS antibiotics: Transcriptional modulation and interactions on the ribosome. Chemistry & Biology 11: 1307–1316. 15380191 10.1016/j.chembiol.2004.07.010

[66] Kohanski MA, DePristo MA, Collins JJ (2010) Sublethal Antibiotic Treatment Leads to Multidrug Resistance via Radical-Induced Mutagenesis. Molecular Cell 37: 311–320. 10.1016/j.molcel.2010.01.003 20159551 PMC2840266

[67] Ezraty B, Vergnes A, Banzhaf M, Duverger Y, Huguenot A, et al (2013) Fe-S Cluster Biosynthesis Controls Uptake of Aminoglycosides in a ROS-Less Death Pathway. Science 340: 1583–1587. 10.1126/science.1238328 23812717

[68] Imlay JA (2015) Diagnosing oxidative stress in bacteria: not as easy as you might think. Curr Opin Microbiol 24C: 124–131. 10.1016/j.mib.2015.01.004 PMC4380616 25666086

[69] Keren I (2013) Killing by bactericidal antibiotics does not depend on reactive oxygen species (vol 339, pg 1213, 2013). Science 340: 1404–1404. 10.1126/science.1232688 23471410

[70] Liu YY, Imlay JA (2013) Cell Death from Antibiotics Without the Involvement of Reactive Oxygen Species. Science 339: 1210–1213. 10.1126/science.1232751 23471409 PMC3731989

[71] Dong TG, Dong S, Catalano C, Moore R, Liang X, et al (2015) Generation of reactive oxygen species by lethal attacks from competing microbes. Proc Natl Acad Sci U S A 112: 2181–2186. 10.1073/pnas.1425007112 25646446 PMC4343105

[72] Imlay JA (2013) The molecular mechanisms and physiological consequences of oxidative stress: lessons from a model bacterium. Nature Reviews Microbiology 11: 443–454. 10.1038/nrmicro3032 23712352 PMC4018742

[73] Penterman J, Nguyen D, Anderson E, Staudinger BJ, Greenberg EP, et al (2014) Rapid Evolution of Culture-Impaired Bacteria during Adaptation to Biofilm Growth. Cell Reports 6: 293–300. 10.1016/j.celrep.2013.12.019 24412364 PMC3941072

[74] West SA, Griffin AS, Gardner A, Diggle SP (2006) Social evolution theory for microorganisms. Nature Reviews Microbiology 4: 597–607. 16845430 10.1038/nrmicro1461

[75] Callaway TR, Edrington TS, Anderson RC, Harvey RB, Genovese KJ, et al (2008) Probiotics, prebiotics and competitive exclusion for prophylaxis against bacterial disease. Anim Health Res Rev 9: 217–225. 10.1017/S1466252308001540 19102792

[76] Kamada N, Chen GY, Inohara N, Nunez G (2013) Control of pathogens and pathobionts by the gut microbiota. Nature Immunology 14: 685–690. 10.1038/ni.2608 23778796 PMC4083503

[77] Gareau MG, Sherman PM, Walker WA (2010) Probiotics and the gut microbiota in intestinal health and disease. Nat Rev Gastroenterol Hepatol 7: 503–514. 10.1038/nrgastro.2010.117 20664519 PMC4748966

[78] Saeidi N, Wong CK, Lo TM, Nguyen HX, Ling H, et al (2011) Engineering microbes to sense and eradicate *Pseudomonas aeruginosa*, a human pathogen. Molecular Systems Biology 7: 521 10.1038/msb.2011.55 21847113 PMC3202794

[79] Liberati NT, Urbach JM, Miyata S, Lee DG, Drenkard E, et al (2006) An ordered, nonredundant library of *Pseudomonas aeruginosa* strain PA14 transposon insertion mutants. Proceedings of the National Academy of Sciences of the United States of America 103: 19931–19931. 10.1073/pnas.0511100103 PMC1413827 16477005

[80] Benoit MR, Conant CG, Ionescu-Zanetti C, Schwartz M, Matin A (2010) New Device for High-Throughput Viability Screening of Flow Biofilms. Applied and Environmental Microbiology 76: 4136–4142. 10.1128/AEM.03065-09 20435763 PMC2897429

[81] Webb JS, Thompson LS, James S, Charlton T, Tolker-Nielsen T, et al (2003) Cell death in *Pseudomonas aeruginosa* biofilm development. Journal of Bacteriology 185: 4585–4592. 12867469 10.1128/JB.185.15.4585-4592.2003 PMC165772

