# Correction: Biofilm Formation As a Response to Ecological Competition

**DOI:** 10.1371/journal.pbio.1002232

**Published:** 2015-08-12

**Authors:** Nuno M. Oliveira, Esteban Martinez-Garcia, Joao Xavier, William M. Durham, Roberto Kolter, Wook Kim, Kevin R. Foster

## Notice of Republication

This article was republished on July 24, 2015 to correct an error in the author byline and article citation. The first author’s name was spelled incorrectly and should be: Nuno M. Oliveira. Please download this article again to view the correct version.
