# Supplementary figures and images for: Biofilm Formation As a Response to Ecological Competition
Source: PLoS Biol. 2015 Jul 9;13(7):e1002191. doi: 10.1371/journal.pbio.1002191 (PMC4497666; doi:10.1371/journal.pbio.1002191)

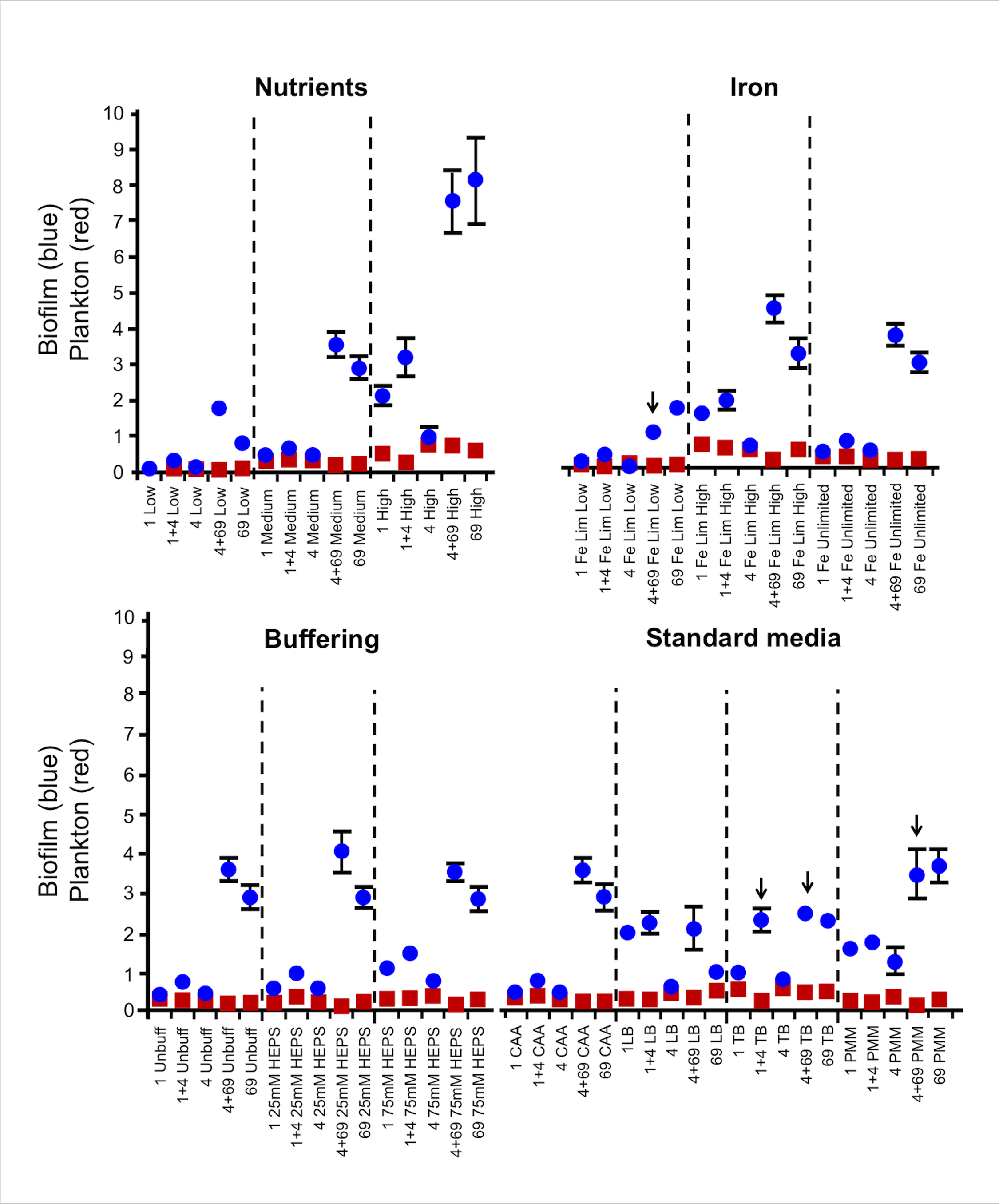

Supplement: S1 Fig — Biofilm formation in single-strain and mixed-strain cultures is shown across a range of conditions. We compared biofilm formation across (i) three nutrient concentrations with casamino acids (CAA) media: 0.1% CAA (low), 0.5% CAA (medium), 2.5% CAA (high); (ii) three iron levels with casamino acids media (100 mg apotransferin + 1 μM FeCl, 100mg apotransferin + 5 μM FeCl, no added apotransferin or FeCl); (iii) three buffering levels with casamino acids media (Unbuffered, 25 mM HEPES, 75mM HEPES); and (iv) four standard media including Luria broth (LB), tryptone broth (TB), Pseudomonas minimal medium (PMM), and casamino acid (CAA) medium. Note that the CAA standard media treatment is replotted in each quadrant for comparison, where it represents medium nutrients, unlimited iron, no buffering, and CAA, respectively. In most cases, strain 4 is outcompeted and represents <5% of the planktonic cells after 24 h. A few exceptions are marked with an arrow where strain 4 is >5% frequency in the planktonic cells. Most notably, the 4 + 69 mixtures marked with an arrow have strain 4 at >90% of planktonic cells, showing how the outcome of competition is strongly influenced by the environment, while the biofilm response is much less so. Error bars indicate the 95% confidence intervals of the mean. Find numerical values in S1 Data. (TIFF) [file pbio.1002191.s002.tiff]

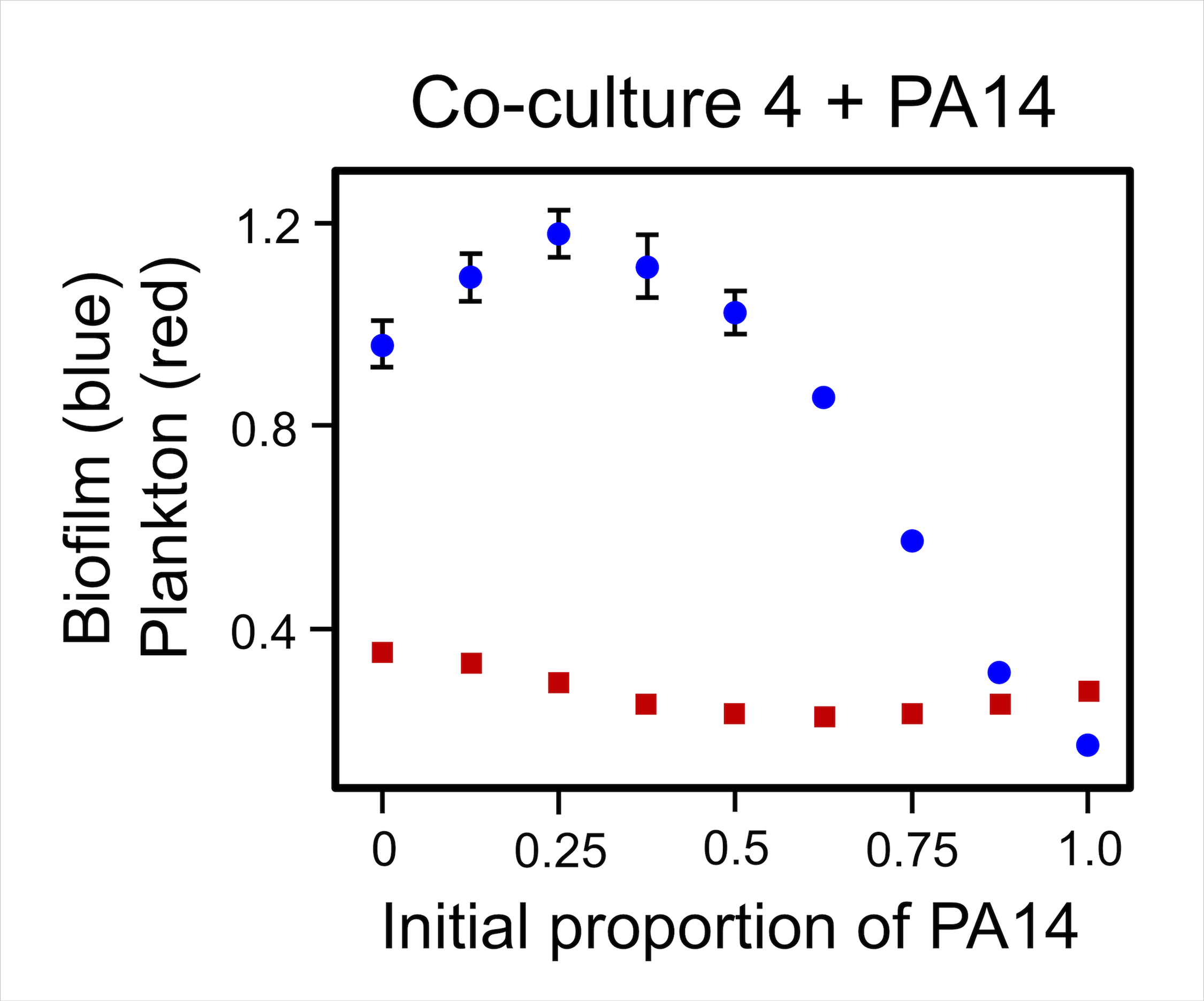

Supplement: S2 Fig — Error bars are 95% confidence intervals of the mean. Find numerical values in S1 Data. (TIFF) [file pbio.1002191.s003.tiff]

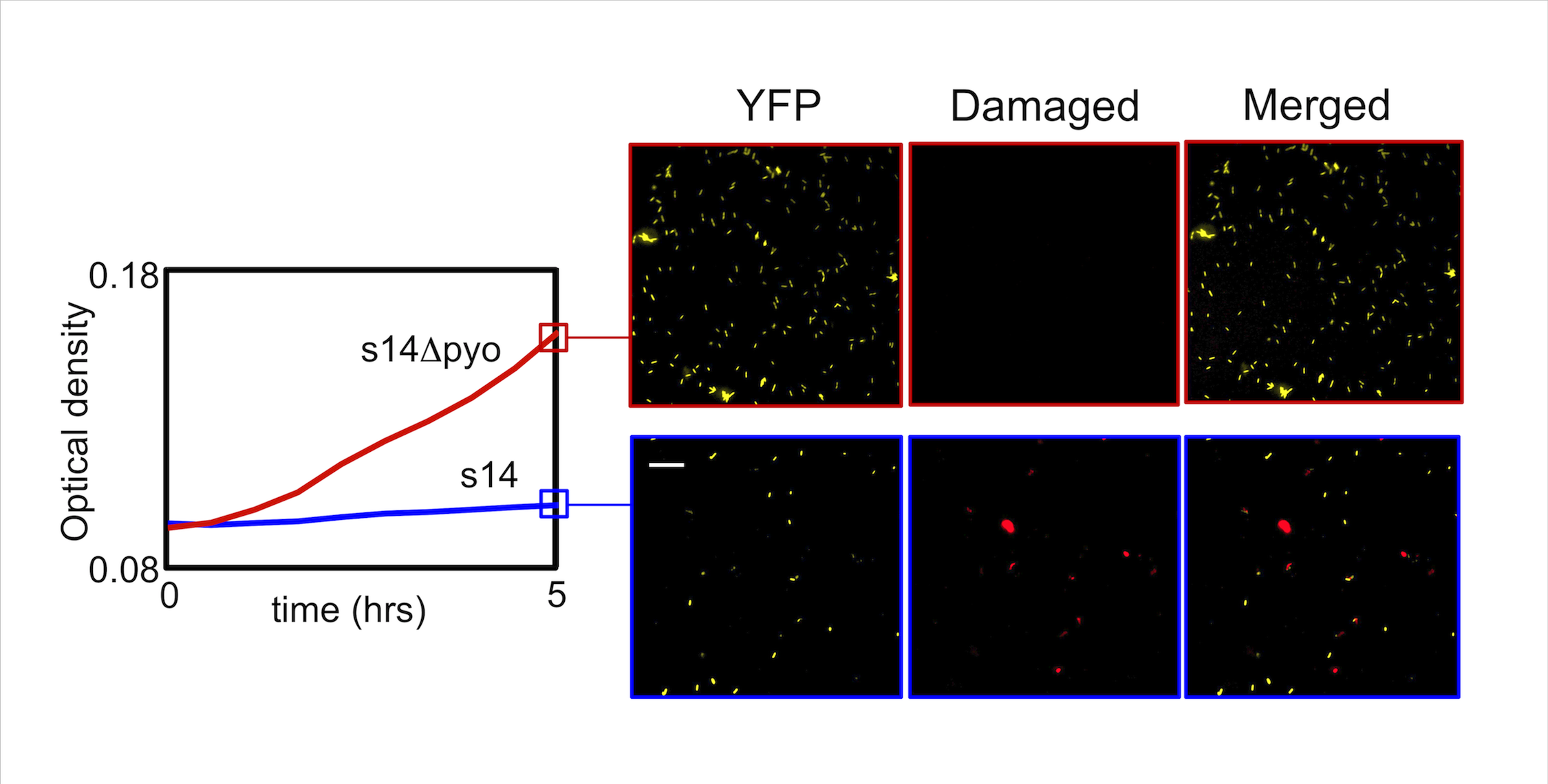

Supplement: S3 Fig — Cell-free supernatant from wild-type (WT) PA14 inhibits the growth of strain 4, as shown in Fig 3. The observed growth lag (left-hand plots) is driven by pyocins, the narrow spectrum antibiotics of P. aeruginosa, and is associated with cell damage (right hand images). After 5 h of growth in the presence of 25% of PA14 cell free supernatant with pyocins (“s14”) or without (“s14Δpyo”) we stained cultures of strain 4 with propidium iodide, and imaged cells using epifluorescence microscopy (see S3 Table for details of PA14 strains). The responder genotype (strain 4) is tagged with a fluorescent protein (YFP), and damaged cells are seen in red. The growth curves on the left are means of eight independent replicates and images on the right are representative images of cell damage in the cultures at the end of the growth period (large red blob is an out-of-focus damaged cell). Images from each fluorescent channel were collected and processed using the same settings to ensure consistency. Scale bar is 20 μm. Find numerical values in S1 Data. (TIFF) [file pbio.1002191.s004.tiff]

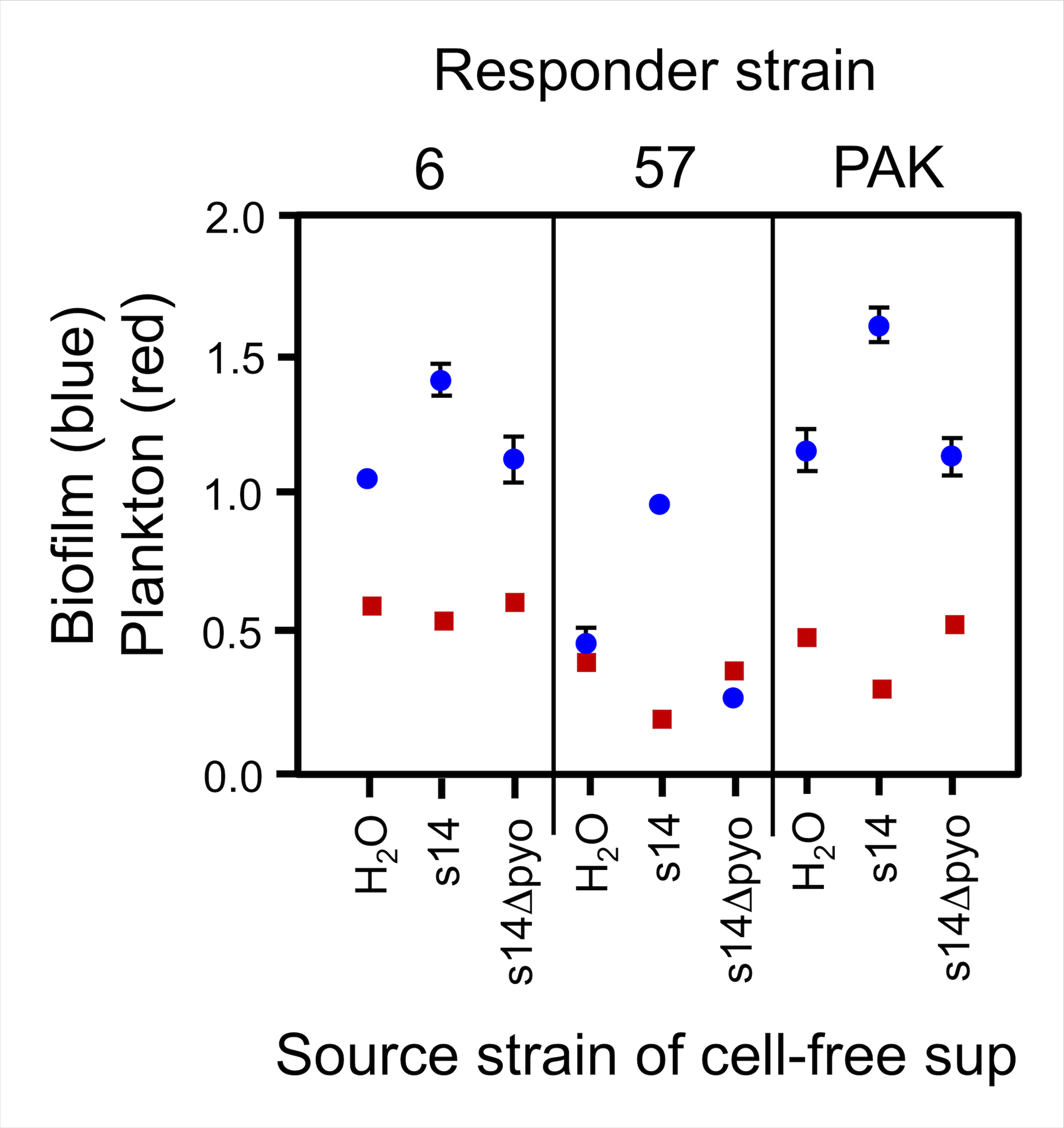

Supplement: S4 Fig — Shown are the effects of cell-free culture supernatants from PA14 and the pyocin-null mutant (14Δpyo) on two natural isolates from Fig 1C. Pyocin-induced biofilm formation is also observed in PAK, a strain known to be susceptible to pyocins from PA14. Error bars are 95% confidence intervals of the mean. Find numerical values in S1 Data. (TIFF) [file pbio.1002191.s005.tiff]

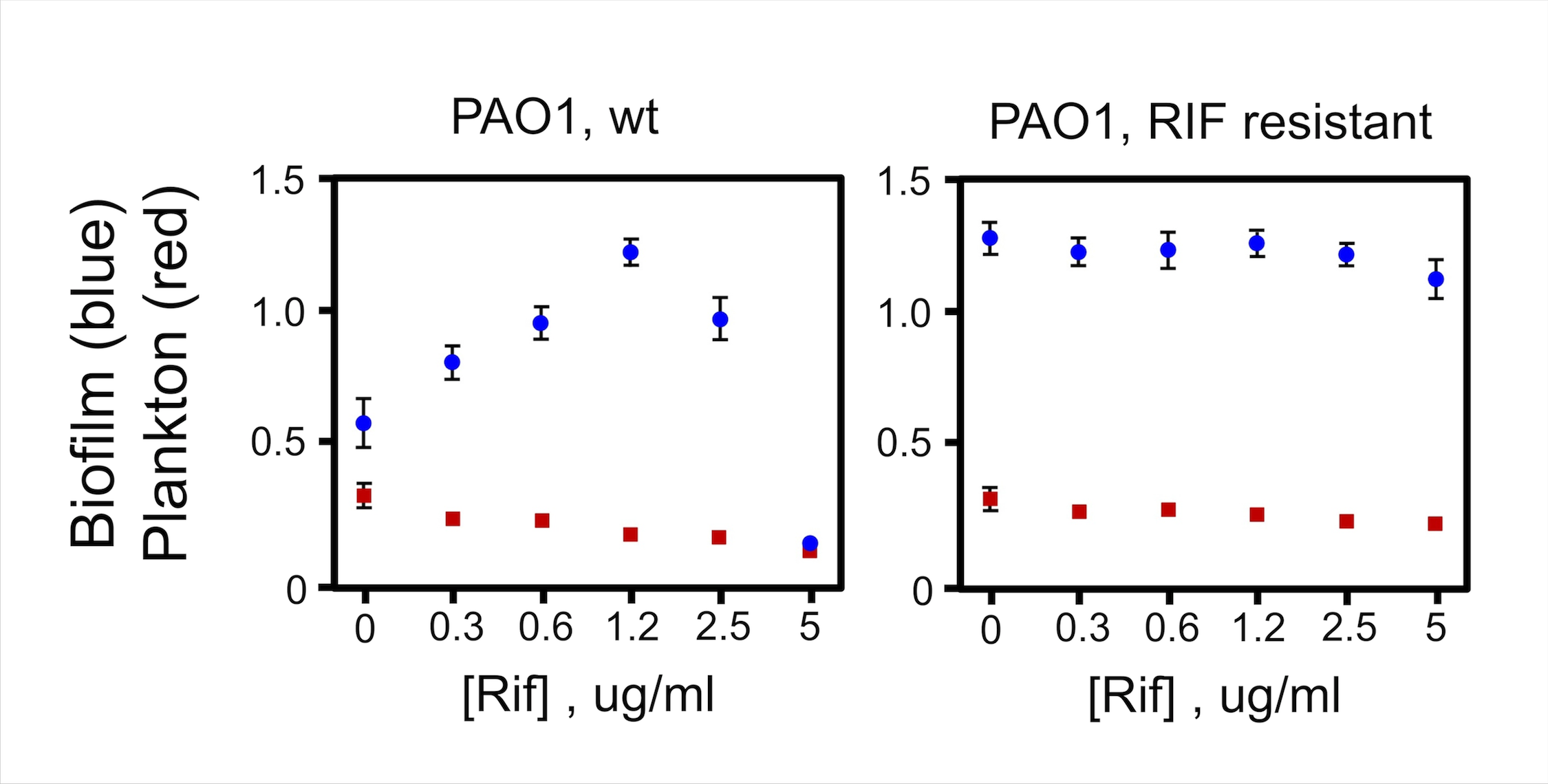

Supplement: S5 Fig — Strain PAO1 exhibits a rifampicin-induced biofilm response, but its rifampicin resistant mutant does not. Error bars are 95% confidence intervals of the mean. Find numerical values in S1 Data. (TIFF) [file pbio.1002191.s006.tiff]

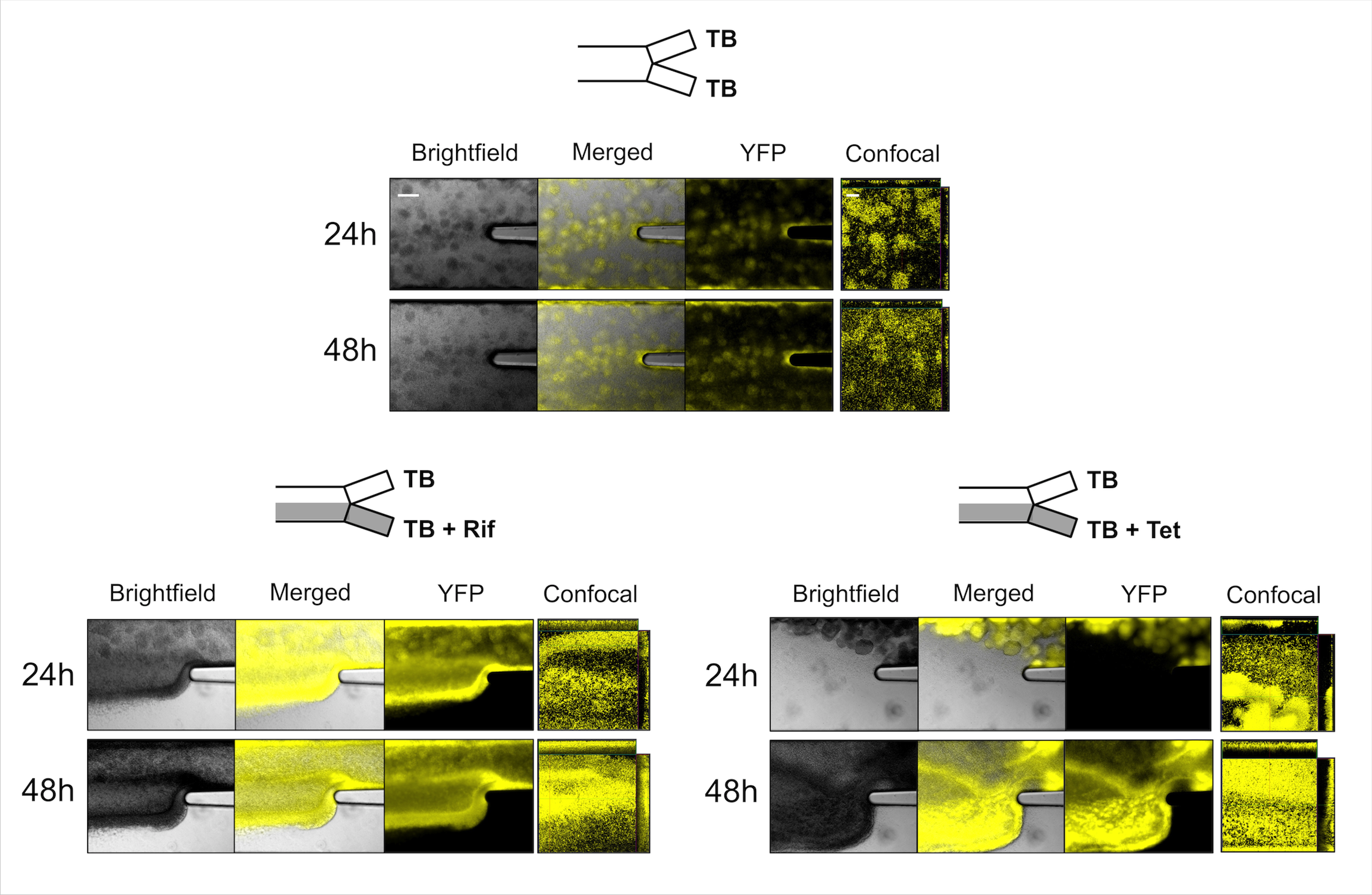

Supplement: S6 Fig — Gradients of rifampicin (Rif) and tetracycline (Tet) drive biofilm accumulation in strain 4 as one finds with gradients of ciprofloxacin (Fig 6). The effect is particularly clear after 48 h of incubation when the biofilm increase is such that blocks the flow. For each treatment we show representative epifluorence microscopy images captured with 10x lens (scale bar is 100 μm for “Brightfield,” “YFP,” and “Merged”), as well as representative confocal images captured with 40x lens (scale bar is 30 μm). Epifluorence images were collected and processed using the same settings to ensure consistency. Confocal images show that the increase in the YFP signal with antibiotic gradients is explained by an increase in both cell density and volume of the biofilm. (TIFF) [file pbio.1002191.s007.tiff]

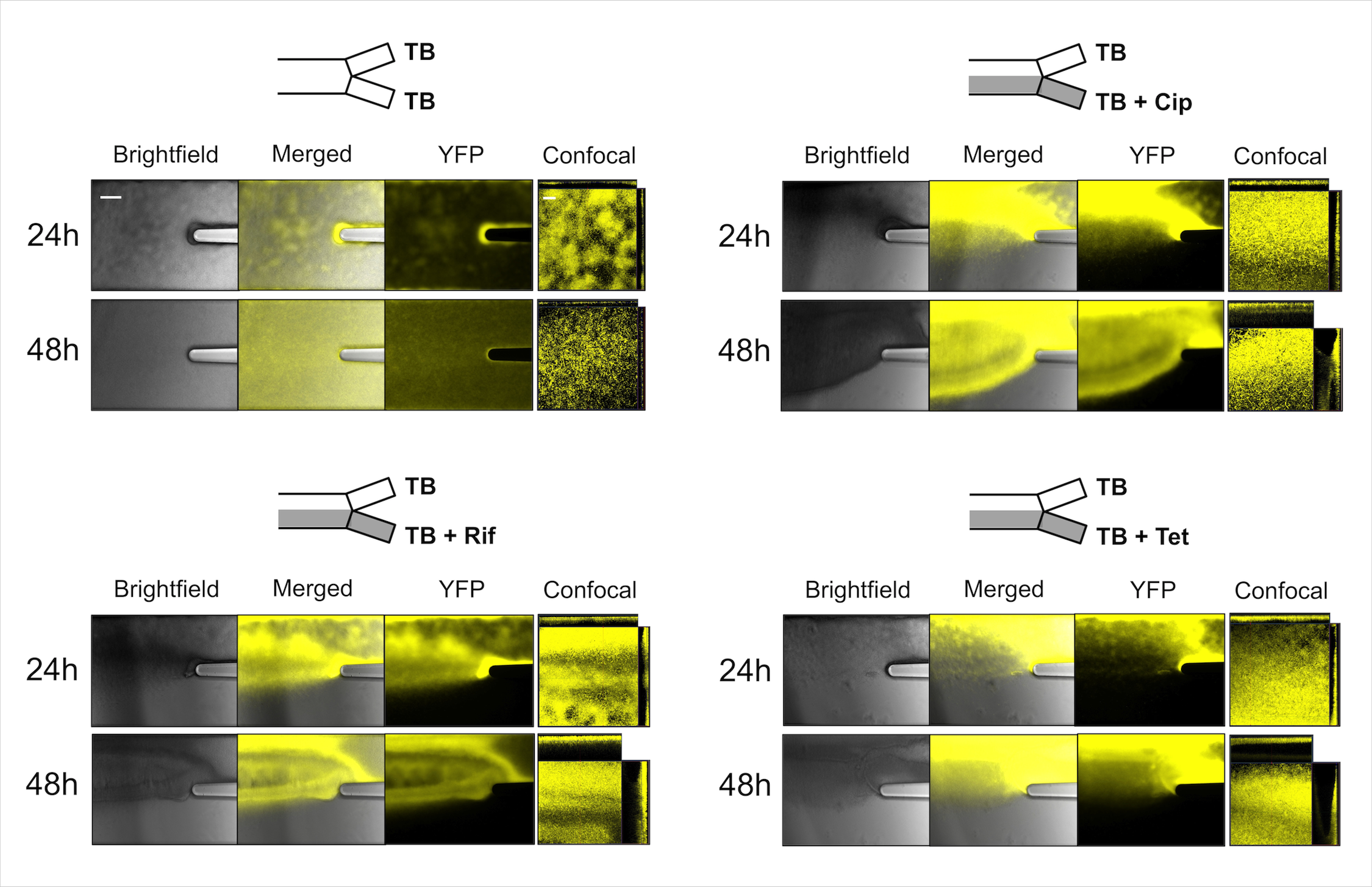

Supplement: S7 Fig — Gradients of ciprofloxacin (Cip), rifampicin (Rif), and tetracycline (Tet) drive biofilm accumulation. The effect is particularly clear after 48 h of incubation when the biofilm increase is such that it can block the flow. For each treatment we show representative epifluorence microscopy images captured with 10x lens (scale bar, 100 μm for “Brightfield,” “YFP,” and “Merged”), as well as representative confocal images captured with 40x lens (scale bar is 30 μm). Confocal images show that the increase in the YFP signal with antibiotic gradients is explained by an increase in both cell density and volume of the biofilm. (TIFF) [file pbio.1002191.s008.tiff]

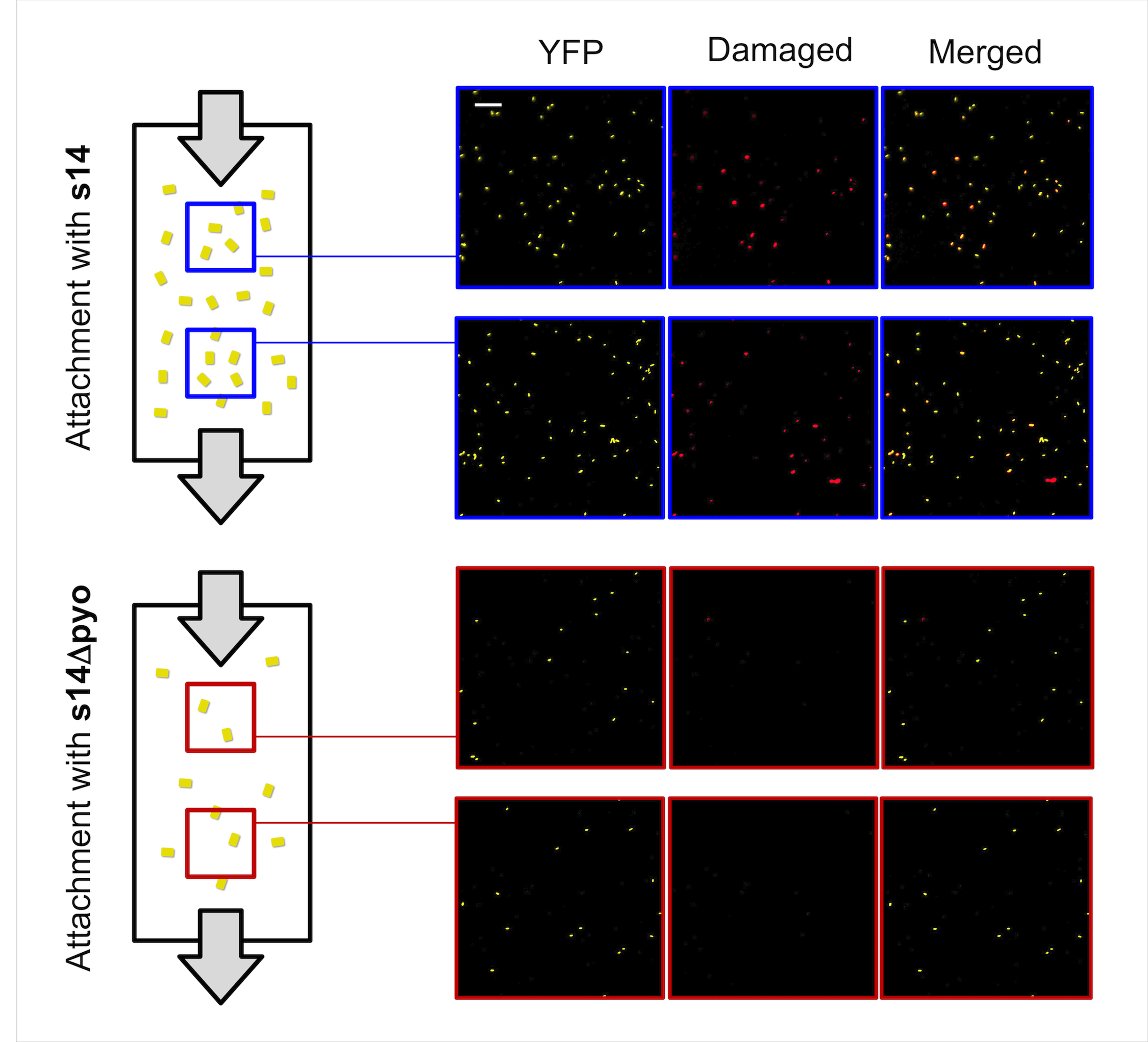

Supplement: S8 Fig — Cell-free supernatant from WT PA14 (s14) promotes surface attachment of strain 4 when compared to supernatant from the respective pyocin-null genotype (s14Δpyo) as observed by two representative regions of each channel. Following the attachment phase we stained yfp-labeled cells with propidium iodide to test for cell damage (red). Images from each fluorescent channel were collected and processed using the same settings to ensure consistency. Scale bar is 20 μm. Experiments were repeated on different days to validate results. (TIFF) [file pbio.1002191.s009.tiff]

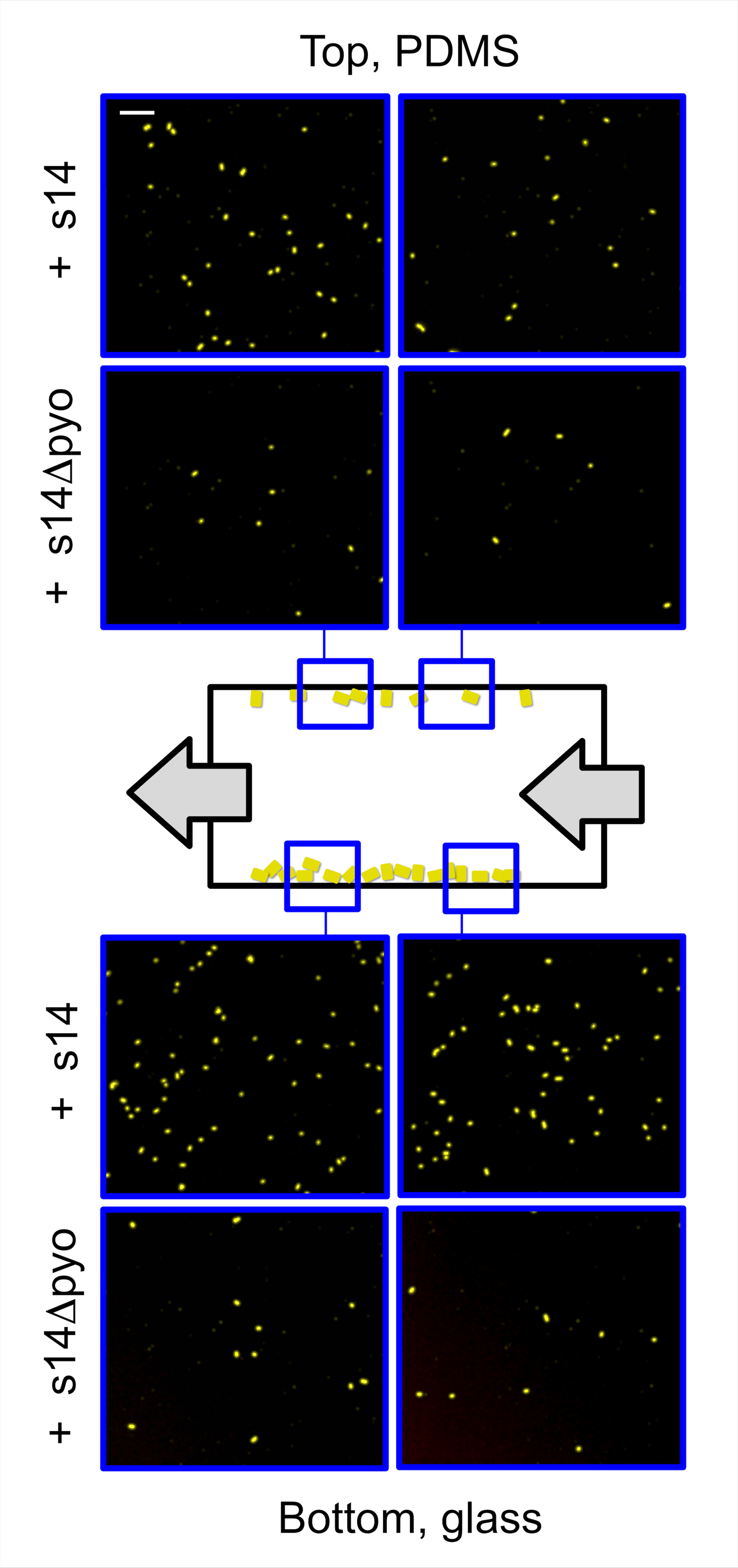

Supplement: S9 Fig — Specifically, we see increased cellular attachment of YFP-labeled strain 4 in the presence of pyocin-containing cell-free supernatant from WT PA14 (s14) on both the bottom (glass) and top (PDMS) of channels, when compared to supernatant from the respective pyocin-null genotype (s14Δpyo). p < 0.05 in unpaired t tests at 5% significance. We show two representative images of each condition. Scale bar is 20 μm. (TIFF) [file pbio.1002191.s010.tiff]

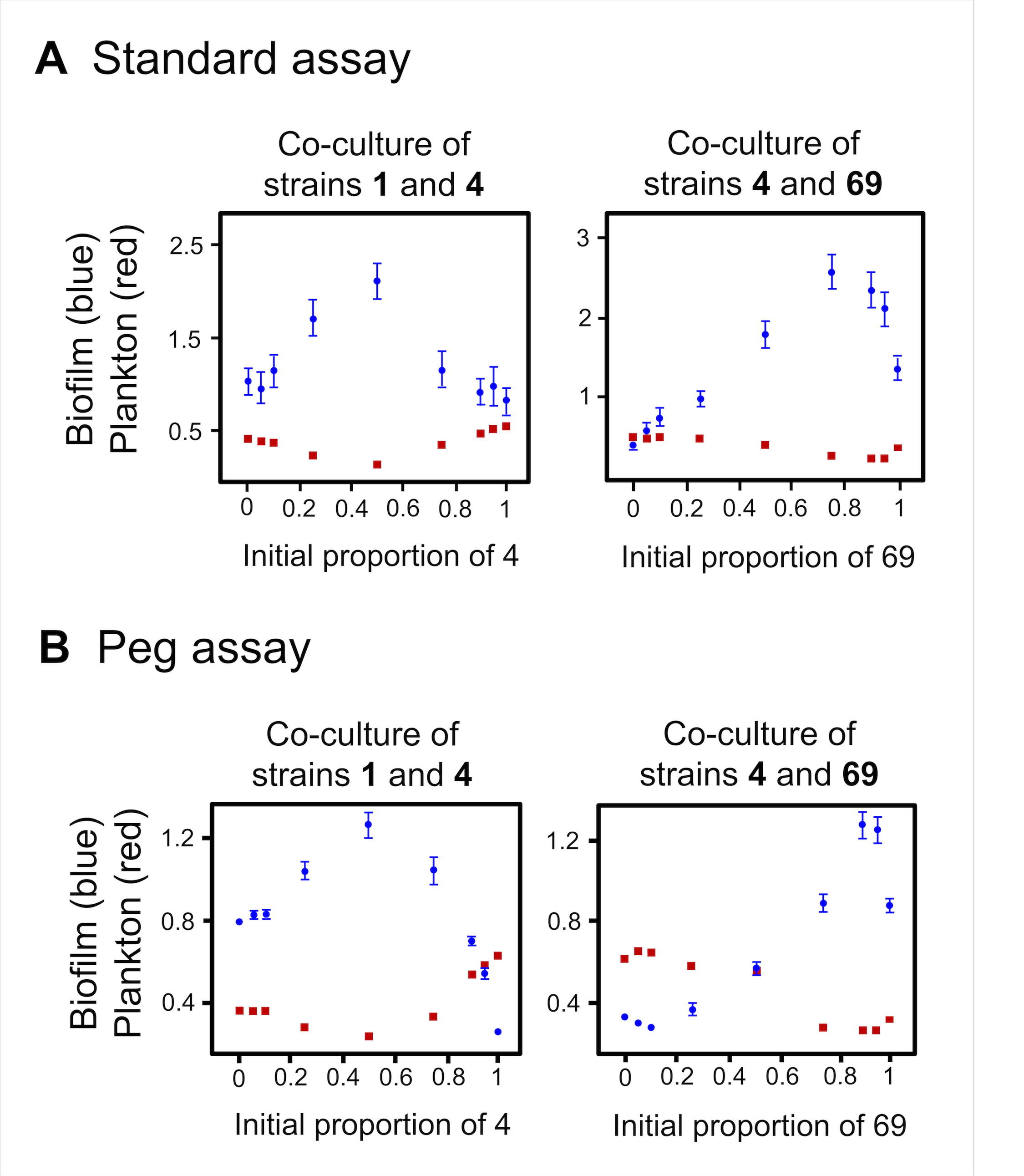

Supplement: S10 Fig — In standard assays, the biofilms measured are those attached to the edge of the wells in microtiter plates. In peg assays, the biofilms measured are those attached to pegs inserted into the wells. Here we show dose-dependent effects of strain mixing on the biofilm response of strains 1 and 4 (left) and 4 and 69 (right). Increased biofilm due to strain mixing is seen in both assays. Error bars are 95% confidence intervals of the mean. Find numerical values in S1 Data. (TIFF) [file pbio.1002191.s011.tiff]

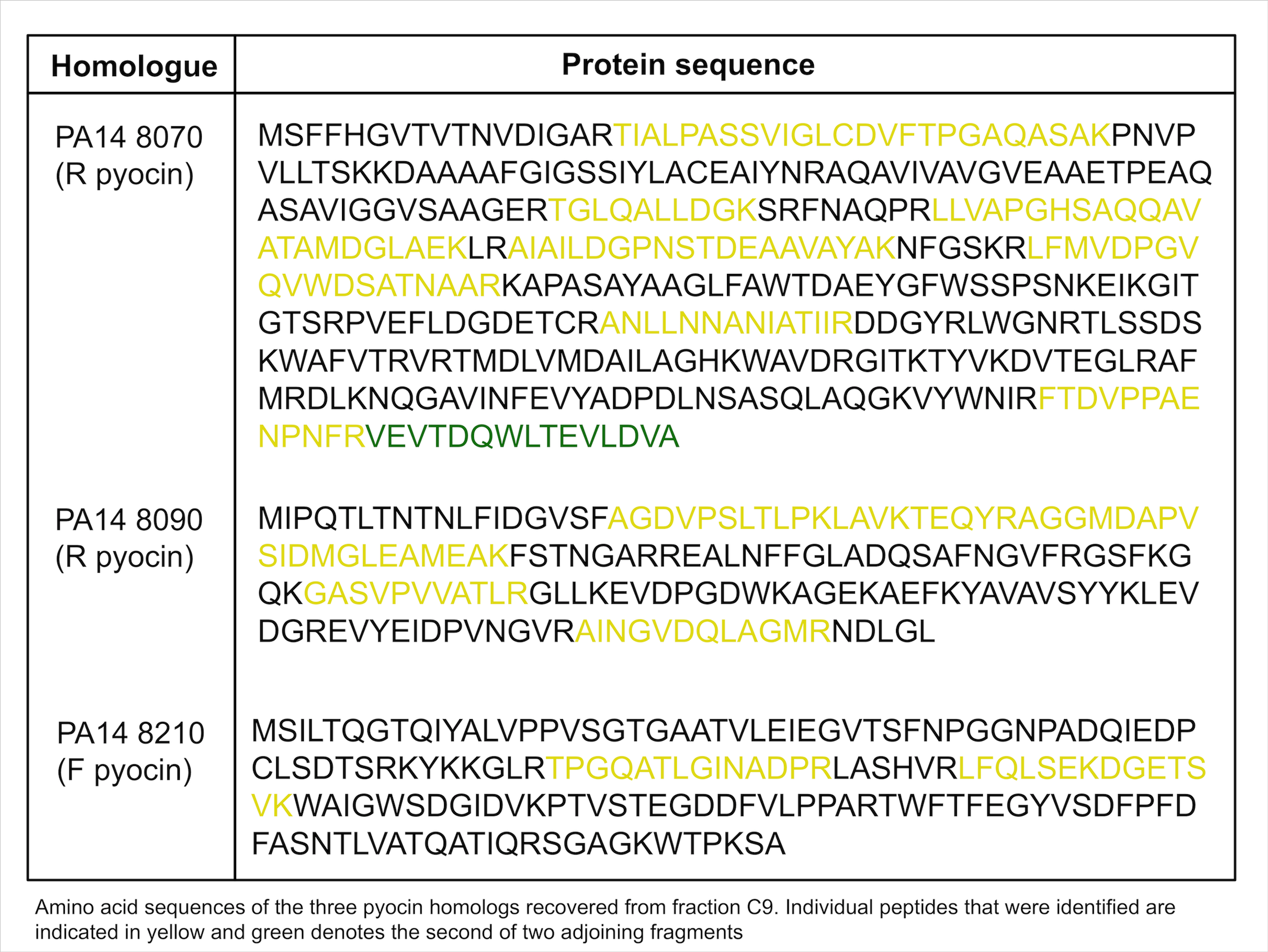

Supplement: S1 Table — (TIFF) [file pbio.1002191.s012.tiff]

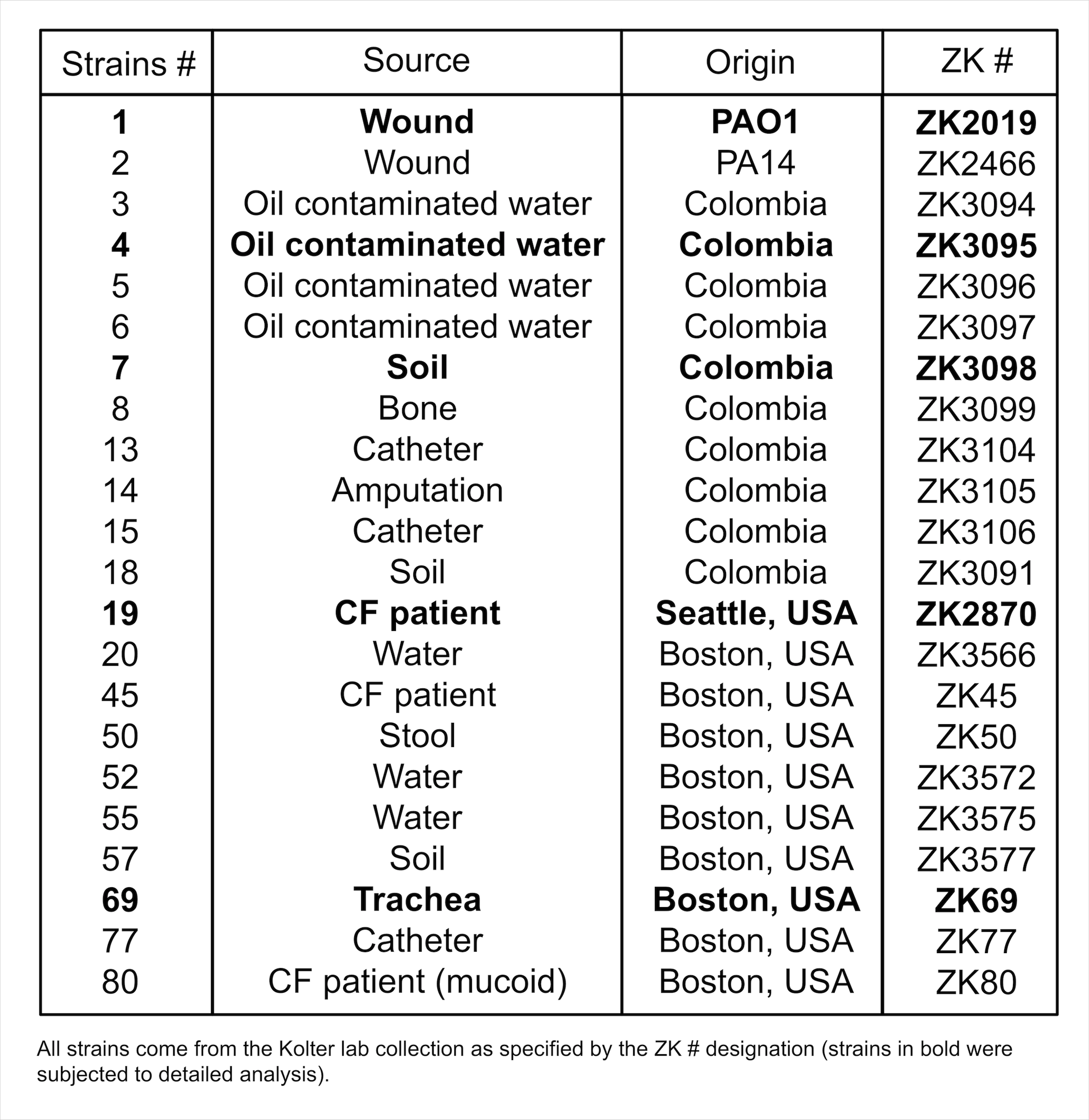

Supplement: S2 Table — (TIFF) [file pbio.1002191.s013.tiff]

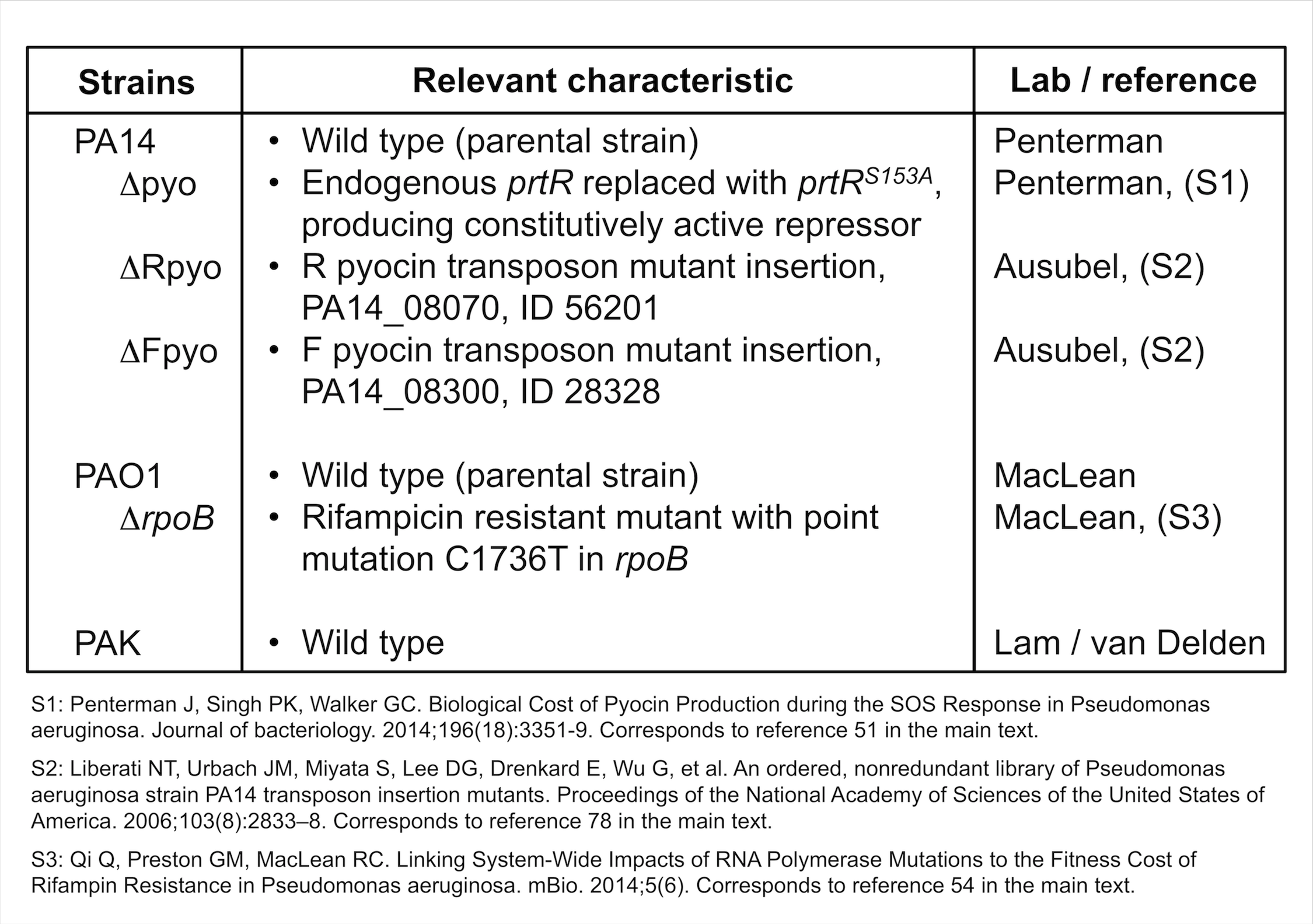

Supplement: S3 Table — (TIFF) [file pbio.1002191.s014.tiff]
